# Generative Autoencoders Coupled to Monte Carlo Simulation Allow Efficient Protein Conformation Sampling

**DOI:** 10.1021/acs.jctc.5c01813

**Published:** 2026-05-25

**Authors:** Jan Beránek, Guglielmo Tedeschi, Vojtěch Spiwok

**Affiliations:** Department of Biochemistry and Microbiology, 52735 University of Chemistry and Technology, Prague 166 28, Czech Republic

## Abstract

Molecular simulations of proteins are well-known to be computationally expensive. Here, we present a new latent-space-based method for modeling protein conformational flexibility at a very affordable computational cost. The method is data-driven and employs an autoencoder-based machine learning model for reversible dimensionality reduction of diverse conformations of the protein studied. Next, samples are selected from the low-dimensional latent space via Monte Carlo sampling. The folding and unfolding of the miniproteins can be sampled in minutes of computational time. We validated the method on four model systems: Tryptophan Cage, nonfolding variant of Tryptophan Cage, Villin headpiece, and human β-2-syntrophin PDZ domain (miniproteins with 20, 20, 35, and 95 residues, respectively). All systems were modeled at an all-atom resolution. Tryptophan Cage and Villin miniproteins show very similar populations of folded/unfolded states sampled by Monte Carlo simulations as the reference MD trajectories calculated by D. E. Shaw Research.

## Graphical abstract

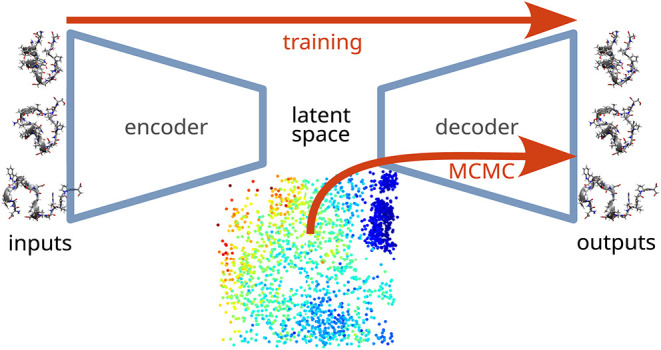

## 1 Introduction

For many years, scientists have been improving methods for studying properties of molecular systems, such as molecular dynamics (MD) simulations, to achieve better effectiveness and computational efficiency.^1^ A lot of effort has recently been invested in improving the accuracy of predictions based on these simulations, for example, by improving existing force fields, therefore obtaining more accurate results for the same computational cost. Another approach to reach more precise predictions is by improving the sampling efficiency by increasing the calculation speed, using general-purpose hardware like GPUs, or even application-specific hardware, e.g., Anton.^2^ Both of these approaches are important to develop and employ—advancements in the development of more precise force fields reduce the systematic errors of predictions of the studied properties, while increased calculation speed allows researchers to run longer simulations, which leads to more thorough sampling and reduces the magnitude of random errors.^3^

Nevertheless, for many systems, it remains challenging to study their dynamics by MD alone. The sampling of important states can be improved by employing one or a combination of enhanced sampling methods. However, many of those may increase the computational cost by themselves (like replica exchange methods), and some require nontrivial design of collective variables (CVs). The design of efficient CVs is especially challenging for complex dynamic processes, such as miniprotein folding, where simple geometric CVs do not suffice. One of the reasons why protein structure sampling with MD can be inefficient is caused by the MD algorithm itself—each subsequent frame of MD trajectory is calculated from the previous one, by evaluating all of the forces acting on each particle as precisely as possible. This means there is a causal relationship between individual frames, which is helpful when studying trajectories of dynamic processes; however, it also complicates statistical analysis of the results.

When the research task requires sampling of many conformations, which should be as diverse as possible, using MD may not be the most-efficient strategy. In such cases, Monte Carlo simulations may be favorable if there is a fast and reliable way to generate new realistic states of the studied systems.

In this work, we present a computationally affordable workflow for protein conformation sampling. It is based on a Monte Carlo simulation combined with generative machine learning. For the generation of protein structures, we use a pretrained generative autoencoder, which fulfills two roles—it reduces the dimensionality of protein conformational space, and also generates structures, some of which were not present in the training data set. The workflow is developed and tested on four model systems—K8A mutant of the TC10b variant of the Tryptophan Cage miniprotein (referred to as TrpCage in the following text), nonfolding variant of the Tryptophan Cage—W6F mutant of the TC5b variant of the Tryptophan Cage (referred to as W6F-TC5b-TrpCage), the Villin headpiece miniprotein and the human β-2-syntrophin PDZ domain.

We demonstrate that the method allows very computationally cheap sampling of the protein conformational behavior. The method is developed to require short wall times from the generation of the training data and autoencoder training to structure generation, energy minimization, and finally MCMC sampling. The limitation of the method is that its speed comes with a small penalty to the precision of the kinetic and thermodynamic parameters, which is lower than for traditional methods. Nevertheless, we show that the method successfully models miniprotein folding for systems up to 35 amino acid residues (the villin headpiece system). For large systems comparable to the PDZ domain or larger, the method is suboptimal at modeling the folding/unfolding processes but can be used for studying less disruptive conformational behavior, allosteric effects, sampling of close-to-native structures usable for ligand docking, collective variable development, etc.

## 2 Methods

The workflow that we present consists of several parts. First, it is necessary to obtain the trajectory of the studied protein that covers as much of the protein conformational space as possible. We show that a relatively short (210 ns) MD trajectory containing the thermal unfolding of the protein can be used for this purpose.

Next, protein structures sampled from the trajectory were used to train an autoencoder. Autoencoders (AEs) are machine learning models designed to allow reversible reduction of dimensionality by encoding complex input data as low-dimensional vectors in the latent space and decoding the original data from this representation.^4^ We used the trained decoder part of the autoencoder to generate new all-atom structures of the studied protein from its latent space representation.

Later, we sampled from the structures generated from the latent space coordinates using Markov chain Monte Carlo (MCMC). A Metropolis-Hastings acceptance criterion is applied to ensure detailed balance of the resulting samples. The whole process is illustrated in Figure 1.

**Figure 1:**
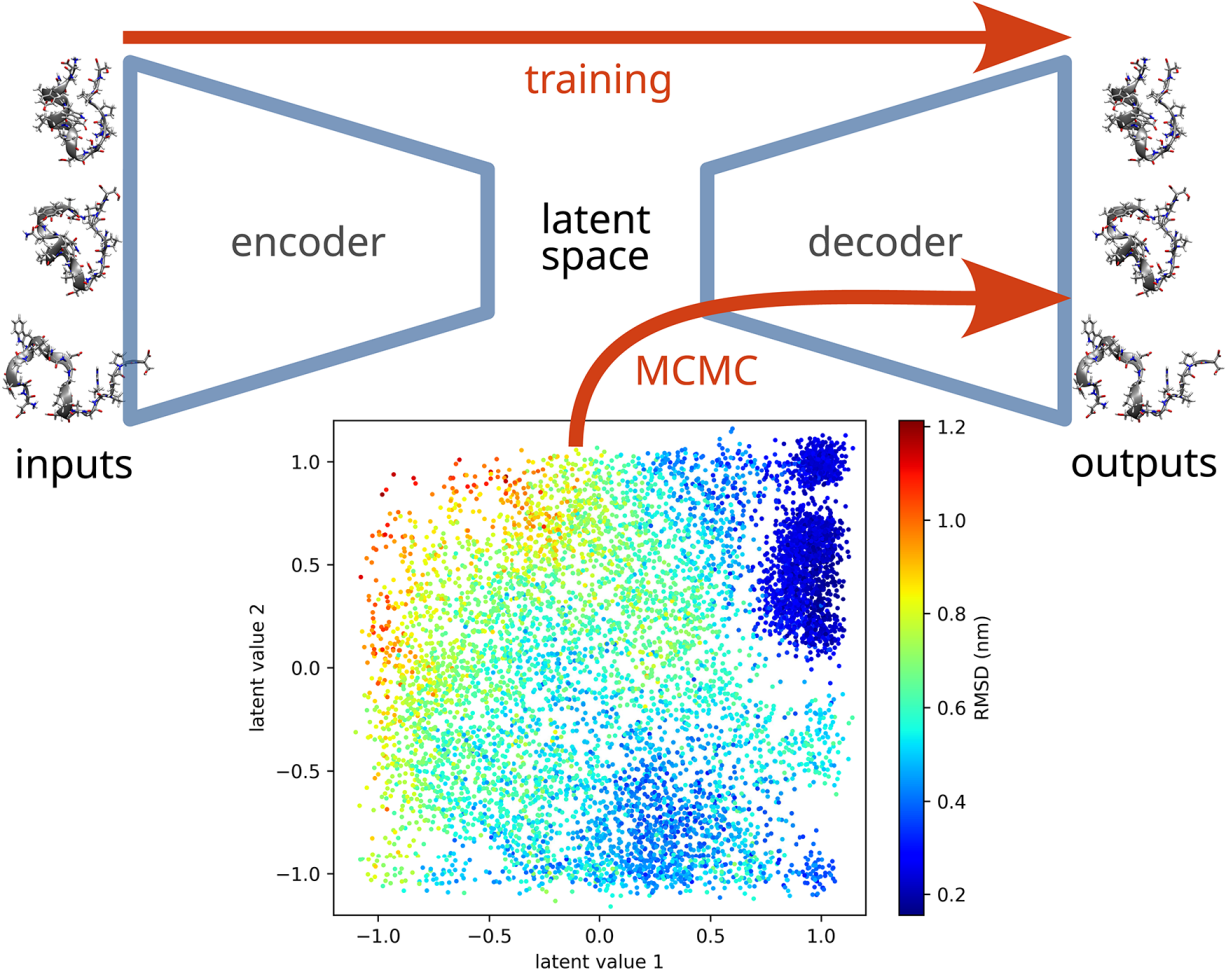
Diagram of the method presented here: First, an autoencoder is trained on (mini)­protein structures. Next, the latent space is sampled and decoder part of the autoencoder is used to generate (mini)­protein structures. These are then accepted or rejected by Metropolis-Hastings MCMC.

### 2.1 Training Data Set Preparation

For the TrpCage and villin headpiece systems, we had long MD trajectories that were kindly provided to us by D. E. Shaw Research,^5^ but we only used them as a reference for evaluation of our method post hoc. In real-world use cases, researchers often do not have the resources to conduct an extensive MD simulation in the first place. Therefore, for all systems, we conducted a series of short MD simulations at high temperatures to sample the process of unfolding of the miniprotein and a variety of its unfolded or misfolded structures. We refer to this procedure as thermal unfolding. The thermal unfolding trajectories we used consist of 210 ns of MD simulations in total. First, a 10 ns long MD was run at 400 K. Four equally spaced frames were sampled from this simulation and were used as starting points for four independent following simulations run for 50 ns at 500 K each. Then, the 400 K simulation was sampled every 1 ps and the 500 K simulations were sampled each 5 ps, resulting in a total of 50,000 frames that were used for training of neural networks. For more technical details, see Supporting Information (SI). In the case of a completely unknown system, AlphaFold^6,7^ models can be used to predict the initial native structure. To validate this approach, we have compared the TrpCage structures sampled during thermal unfolding to the reference MD trajectory by D. E. Shaw.^5^ For over 10,000 structures sampled from the reference MD trajectory, we calculated the Cα-RMSDs from the most similar structure in the training data set. The maximum Cα-RMSD value for such a structure pair was under 0.38 nm, with the median of the values ≈ 0.26 nm; therefore, we conclude that the thermal unfolding strategy samples almost all relevant miniprotein structures in much shorter computational time but at high temperature. For more details on this analysis, see the SI.

### 2.2 Generative Autoencoder

Training data—scaled Cartesian coordinates of all protein atoms, in total 50,000 conformations—were used to train the autoencoder. 20 % of the data set was used as a test data set used to evaluate the performance of models. The autoencoder architecture has been built in Keras 3.4.1 ^8^ using the TensorFlow^9^ backend.

The neural network used for TrpCage miniprotein (K8A mutant of the variant TC10b,^5^ reference PDB: 2JOF^10^) had an input layer with dimensionality equal to the number of all protein atoms × 3, followed by an encoder part consisting of several feed- forward layers, with 512, 128, 32, and 8 neurons, respectively, and the last “latent space” layer with two neurons. The input conformation was fitted to the reference PDB structure^5^ by minimizing the Cα-RMSD. The reference structure was then subtracted from the input so the autoencoder only learns the internal motions of the studied miniprotein. The coordinates of the miniprotein atoms were also scaled to an interval between 0.0 and 1.0 before being submitted to the neural network. The reverse operation was done on the output from the decoder. The output of the latent space layer has a Gaussian noise added to it to regularize the latent space, similarly to how variational autoencoders^11^ work. This was done by adding a random number sampled from the normal distribution, centered in the encoder output, with a standard deviation of 0.05.

During training, the latent representation of the data is used as an input for the decoder part, consisting of dense layers that have 8, 32, 128, and 512 neurons, respectively, mirroring the architecture of the encoder. The last dense output layer has the number of neurons equal to the dimensionality of the input data, number of all protein atoms ×3. Then, the coordinates of the reference structure of the protein are added to the decoder output, and the result of this operation is the output of the whole model. This means that the autoencoder does not encode and decode the whole protein, but only the differences in positions between the reference structure and the encoded one, which improves the scalability of the method to larger systems. Layers with 128 or more neurons (except for the output layer) had batch normalization^12^ applied to their outputs. All layers had the Exponential Linear Unit (ELU) function applied to their output, except for the latent space layer, which had the hyperbolic tangent function applied to its output instead, and the decoder output layer, which used no activation function.

The autoencoder used for the nonfolding W6F-TC5b-TrpCage had the same architecture as the AE for TrpCage. There is no stable folded structure for this TrpCage variant, so as a reference structure for AE and for calculating Cα-RMSD purposes, we used a homology model structure based on the TrpCage reference PDB structure.

The autoencoder used for the villin headpiece (reference PDB: 1YRF^13^) miniprotein was built analogically; it used only more layers. The encoder part comprised layers with 2048, 1024, 512, 128, 32, and 8 neurons, respectively, followed by the latent space layer with 2 neurons. The decoder part of the model had the same number of neurons in layers as the encoder in the opposite order.

The autoencoder used for the human β-2-syntrophin PDZ domain (reference PDB: 2VRF^14^) was built analogically, using layers with 3072, 1024, 512, 128, 32, and 8 neurons, respectively, followed by the latent space layer with 2 neurons and with the decoder mirroring the encoder.

All models were trained using the gradient descent algorithm Adam.^15^ Initially, the models were trained using the mean square error loss function between the original and reconstructed Cartesian coordinates of atoms for 100 epochs. Then, weights in the encoder part were fixed and decoder weights were fine-tuned by training for 5 epochs using the mean square error of all interatom distances. Finally, decoder weights were further fine-tuned by training for 20 epochs using the mean square error of inverse values of all interatom distances.

### 2.3 Markov Chain Monte Carlo Simulations

First, to enhance the performance of the MCMC simulation, it is possible to calculate the protein structures by the decoder in advance, as the decoder is deterministic. The structures were precalculated by the decoder, from combinations of input values equally spaced over the latent space on a 64 × 64 grid ranging from −1.25 to 1.25 for both latent space coordinates. We recommend the users of the method to use sufficiently high resolution grids. The number of bins should be at least the latent space size divided by the standard deviation of the regularizing random noise added to the encoder outputs during the autoencoder training. In our case, this is calculated as 2.5/0.05 = 50, we have used slightly finer resolution 64. To validate this approach, we run MCMC simulations of the TrpCage system using multiple grid resolutions and show the results in the SI.

We developed a special protocol for the potential energy minimization of the decoded structures using the OpenMM library. The protein topology was loaded from the reference PDB structures. The AMBER14 ^16^ force field was used for the protein atoms, and the implicit solvent GBn2 ^17^ was used. Two minimization algorithms were used in the protocol: The potential energy was minimized by the steepest descent algorithm until all of the forces acting on the atoms were smaller than 400 kJ/mol/nm. If the steepest descent algorithm failed to find a local minimum in a predefined number of steps, the initial structure was briefly minimized by the OpenMM default minimization algorithm L-GFBS until all forces were smaller than 10,000 kJ/mol/nm, and the resulting structure was further minimized by steepest descent until all of the forces acting on the atoms were smaller than 400 kJ/mol/nm. For more details on potential energy minimization, see SI. The generated and minimized structures were exported and visually checked for the presence of clashes or bad contacts between atoms. The minimized structures were saved, together with their potential energies.

Then, the MCMC simulation was conducted. The initial structure was selected from the array of precalculated structures from latent space coordinates where folded structures from the training data set were encoded.

Structures and energies for MCMC attempts were selected as follows: At the first and then at every *f*-th step, a random direction was drawn from uniform distribution in the two-dimensional space. Then, for the *f* number of steps, new latent space coordinates were sampled at a distance *d* in the drawn direction from the latent space coordinates of the last structure accepted by the Metropolis-Hastings acceptance criterion. At these new coordinates, the potential energy of the system was estimated by linear interpolation (by “linear” method of Regular Grid Interpolator from SciPy^18^ library) based on the potential energies of the closest structures on the precalculated grid. The interpolated potential energy was then compared to the last accepted energy and was accepted or rejected based on Metropolis-Hastings acceptance criterion (with temperature 290 K, 290 K, 360 or 300 K for the TrpCage, W6F-TC5b-TrpCage, Villin and PDZ domain, respectively).

After reaching the *f* number of attempts since the last random change in direction, the last accepted interpolated potential energy and the structure corresponding to the closest point on the precalculated grid were saved and concatenated into the output file using the OpenMM^19^ library. Then, a new random direction for future attempts was selected, and this process was repeated a specified number of times. The MCMC algorithm is illustrated in Figure 2.

**Figure 2:**
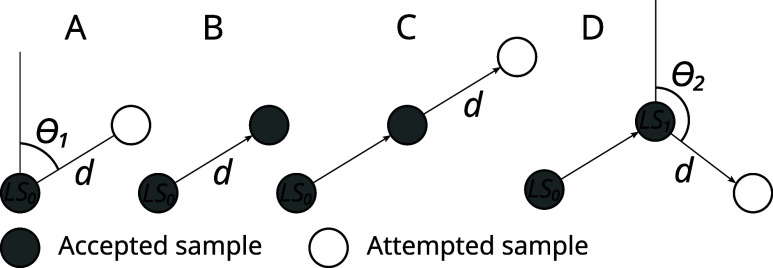
Diagram of an example of the MCMC algorithm for *f* = 2. (A) First, initial position on the latent space LS_0_ is selected and random direction θ_1_ is drawn from uniform distribution. Attempt sample is determined at the point in the direction θ_1_ at the distance *d* from LS_0_. (B) Attempt sample is evaluated by Metropolis-Hastings criterion and in this example, it is accepted. (C) New sample is determined as the point in the direction θ_0_ in the direction *d* from the last accepted sample. This time it is not accepted. (D) After *f*-th step, the last accepted sample LS_1_ is recorded into the output file. A new direction θ_2_ is drawn. New attempt samples will be selected in the θ_2_ direction and at the *d* distance from the last accepted sample.

The *f* is a parameter which can be adjusted—with higher values (*f* in the order of thousands), the exploration of the latent space and corresponding protein conformations is more likely to escape the potential energy minimum of the folded conformation, but the simulation takes more computational time to generate new frames for the MCMC samples, as only every *f*-th frame is recorded. Lower values of *f* (in the order of hundreds) lead to more erratic and thorough exploration of the latent space, but the system is less likely to escape the potential energy minima of the latent space.

The parameter *d* influences the acceptance ratio of the new energies in the Monte Carlo algorithm. Smaller values of *d* increase the acceptance ratio and can help the system overcome high potential-energy barriers by dividing the potential energy gradient into smaller individual steps. The effect of the values of parameters *f* and *d* is demonstrated by running several MCMC simulations of the TrpCage system with various combinations of the parameters. The comparison of the results is in the SI, Figure S4. For new systems, we recommend the users of the method to set the *f* parameter to be rather small, as long as the system still explores the latent space efficiently, and the parameter *d* to be rather large while allowing a reasonable acceptance ratio of the MCMC process.

For the MCMC simulations we present in Results, parameters *f* and *d* were selected by a quick trial-and-error exercise with the goal of obtaining a reasonable Metropolis-Hastings acceptance ratio between 30 and 60% and also observing fast exploration of different regions of the latent space, promising to simulate several unfolding/folding conformational changes during the MCMC simulation. When determining a well-working combination of the *f* and *d* parameters, we started with *f* set to 1 and *d* set to 0.01. We first adjusted the *d* to get the MCMC acceptance ratio between 30 and 60%. If the system failed to explore LS areas outside of the starting folded minimum, we increased the *f* parameter and adjusted the *d* parameter as in the first step. These steps were repeated until the simulation was able to explore the LS efficiently.

The code used in this work is available at https://github.com/Jan8be/aemcmc.

## 3 Results

We show that a relatively simple probabilistic autoencoder is able to model the conformational flexibility of miniprotein foldings and can be utilized as a powerful dimensionality reduction technique from 2D latent space to all-atom resolution. Generated structures can be used to calculate trajectories with the Metropolis-Hastings Markov chain Monte Carlo algorithm. The main advantage of this setup is that the training data set with high conformational diversity can be generated quickly at high temperature (500 K), and these conformations can then be sampled by an autoencoder and MCMC at a temperature relevant to biological processes.

### 3.1 Tryptophan Cage

The autoencoder for the K8A-TC10b variant of the TrpCage miniprotein was trained as described in the Section 2. Here, we demonstrate the ability of the autoencoder to reversibly reduce the dimensionality of all-atom protein structures onto a two-dimensional latent space. Figure 3 depicts structures from the training data set projected into a 2D latent space by the trained encoder part of the model and selected conformations of the miniprotein decoded from hand-picked coordinates from this latent space.

**Figure 3:**
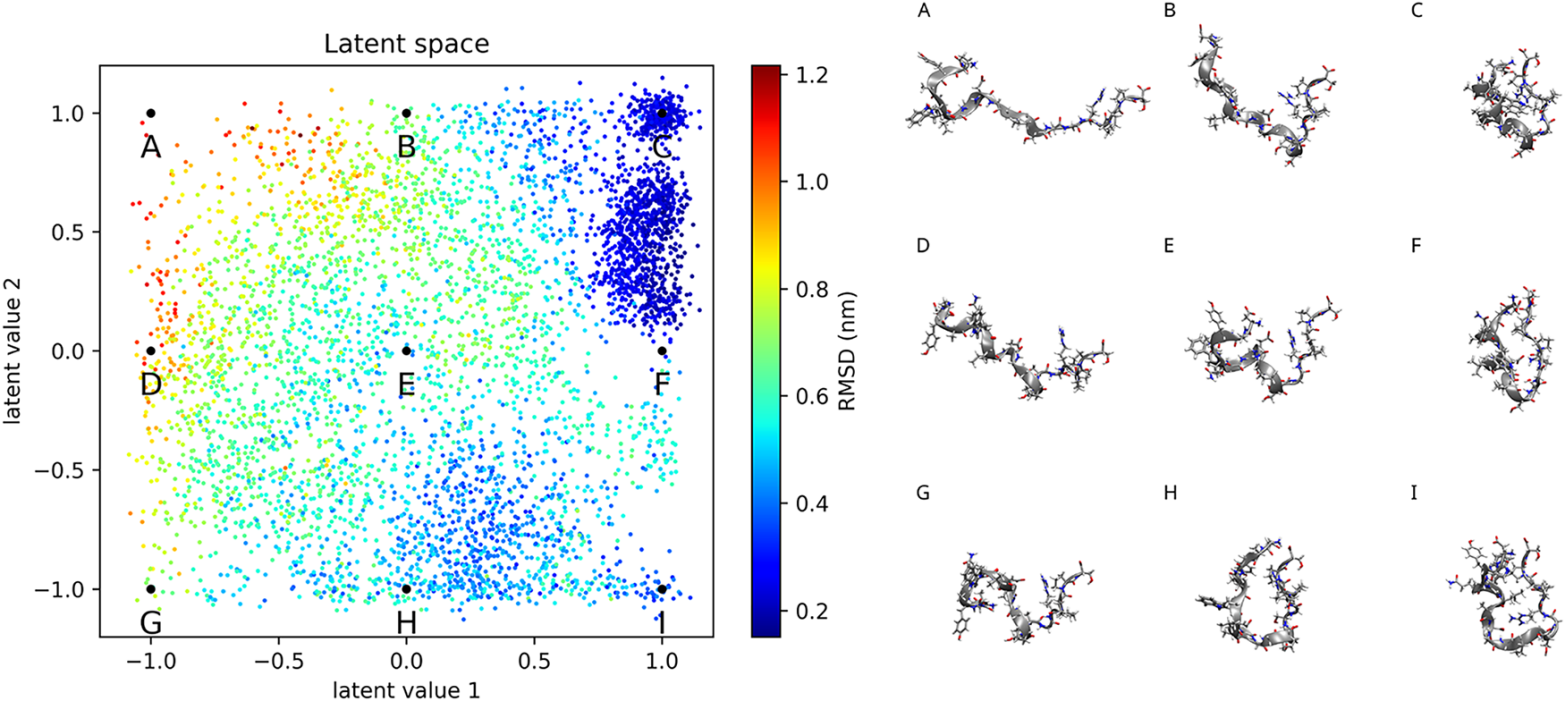
Left: Latent space of TrpCage structures encoded by trained model. Color depicts Cα-RMSD of the encoded protein structures from the folded reference PDB structure. Right: TrpCage structures decoded from hand-picked coordinates from the latent space depicted by letters A–I.

We have also tested several data set sizes for the model training on the TrpCage system. Data sets compiled from the structures sampled from the thermal unfolding trajectories of TrpCage had sizes of 210,000, 50,000, and 10,000 structures, respectively. All data sets were split into a training set (80%) and a validation set (20%). Learning curves for the three data set sizes are shown in Figure 4. Quality of the structures generated by the decoder was assessed by calculating potential energies of 500 randomly selected structures from the data set, which were encoded and reconstructed by models after each part of the training/fine-tuning process for each data set size. The comparison of the distributions in the energies of the reconstructed structures is shown in Figure 5. We did the same analysis of the learning curves and the potential energies of the reconstructed structures of the autoencoder used for the PDZ domain system. The results were analogous to those of the TrpCage system and are shown in the SI, Figures S6 and S7. We assume that for the villin headpiece system, the training would behave analogously, as its size is between the TrpCage and PDZ domain systems we have tested.

**Figure 4:**
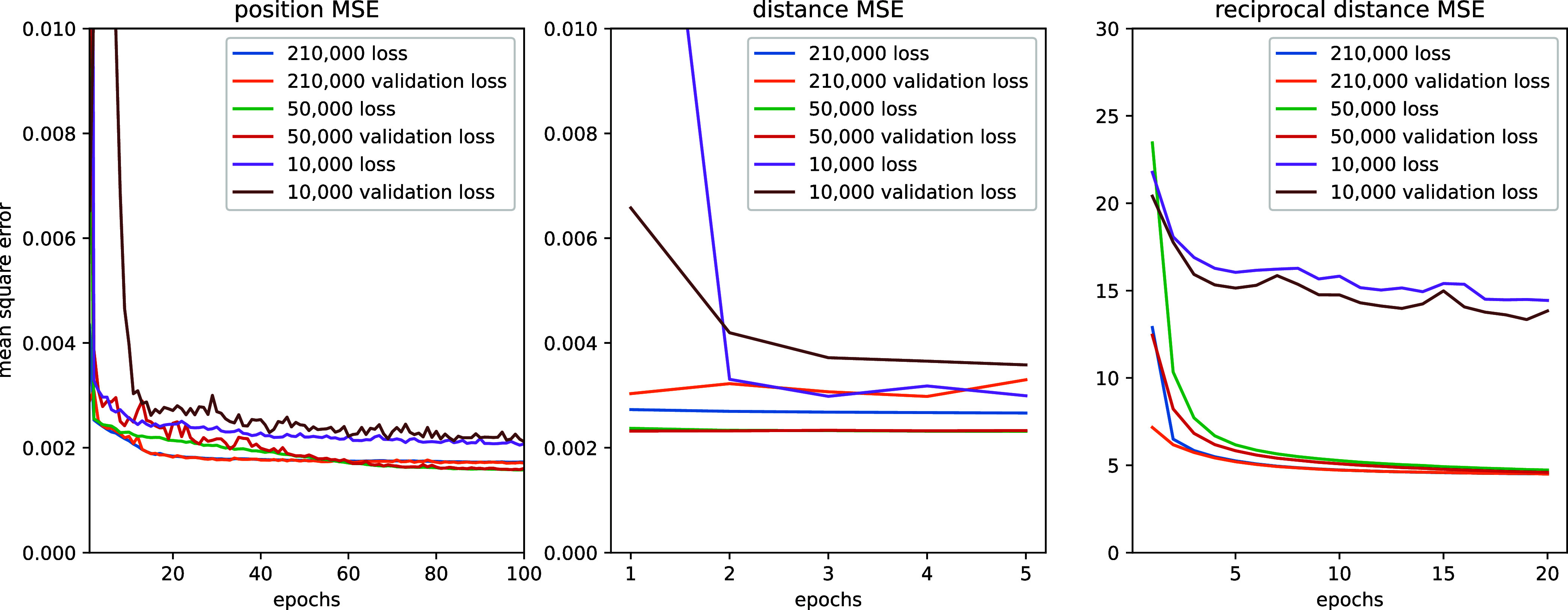
Comparison of learning curves for three different data set sizes.

**Figure 5:**
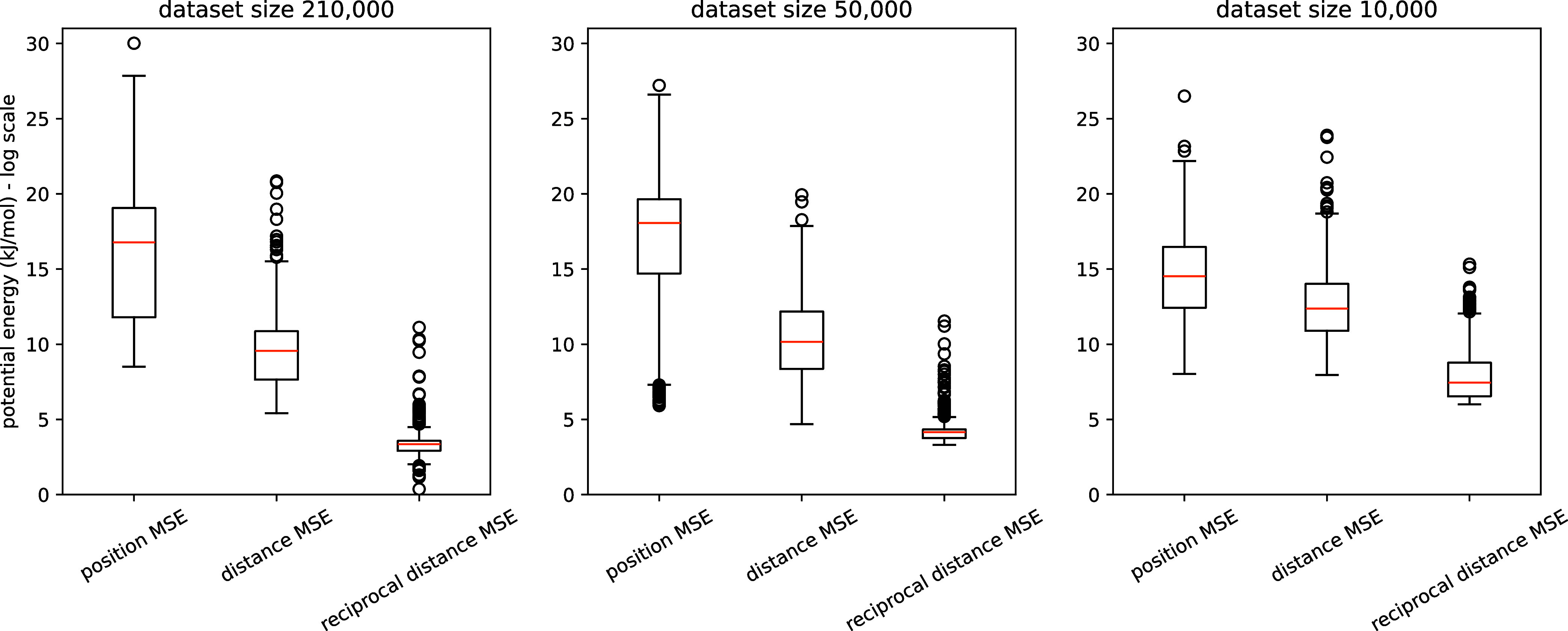
Distributions of potential energies of structures reconstructed by models trained on different data set sizes and after different training/fine-tuning treatments.

Prior to the MCMC itself, 4096 TrpCage configurations were calculated by the decoder. The inputs were latent space coordinates on a 64 × 64 grid ranging from −1.25 to 1.25. Structures had their potential energy minimized in an implicit solvent as described above to mitigate potential bad contacts that may be present in the generated structures, especially in the areas of latent space with less density of training data. The potential energies of the prepared conformations are visualized in Figure 6. MCMC simulation was conducted as described in the Section 2, with parameters *f* = 200 and *d* = 0.001, starting from the folded conformation of TrpCage. The temperature for MCMC was set to 290 K, the same temperature as used by the D. E. Shaw Research,^5^ to allow for direct comparison of our samples with the published MD trajectory. In total, 5 million attempts of the Metropolis-Hastings sampling were conducted. In total, 2,951,342 structures were accepted, so the acceptance ratio was approximately ∼59%. Since samples were collected in every *f*-th frame, this resulted in a 25,000 MCMC samples, which are visualized in Figure 6. The MCMC samples contained 10 unfolding and 9 folding events (see Figure 7). We compared the relative populations of structures based on their Cα-RMSD from the reference structure in our MCMC simulation with the populations in the long MD simulation published by D. E. Shaw Research.^5^ The comparison is visualized in Figure 8. We observe that the two-well FES has the barrier between folded and unfolded state slightly lower than in the reference simulation. We also compared the reference and sampled populations based on their radius of gyration and α helix content (as number od residues forming a helix according to Dictionary of protein secondary structure (DSSP)^20^ calculated using MDTraj^21^ Python package), resulting free energy surfaces are visualized in Figure 9. We observe that both free energy surfaces from MCMC simulation are very similar to corresponding reference.

**Figure 6:**
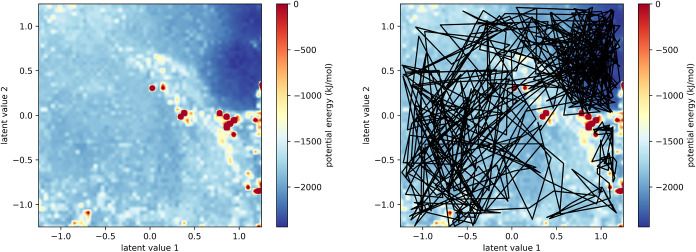
Left: Interpolated potential energy surface of TrpCage latent space. Right: MCMC samples visualized over the latent space. Only every 50th frame is shown for better clarity.

**Figure 7:**
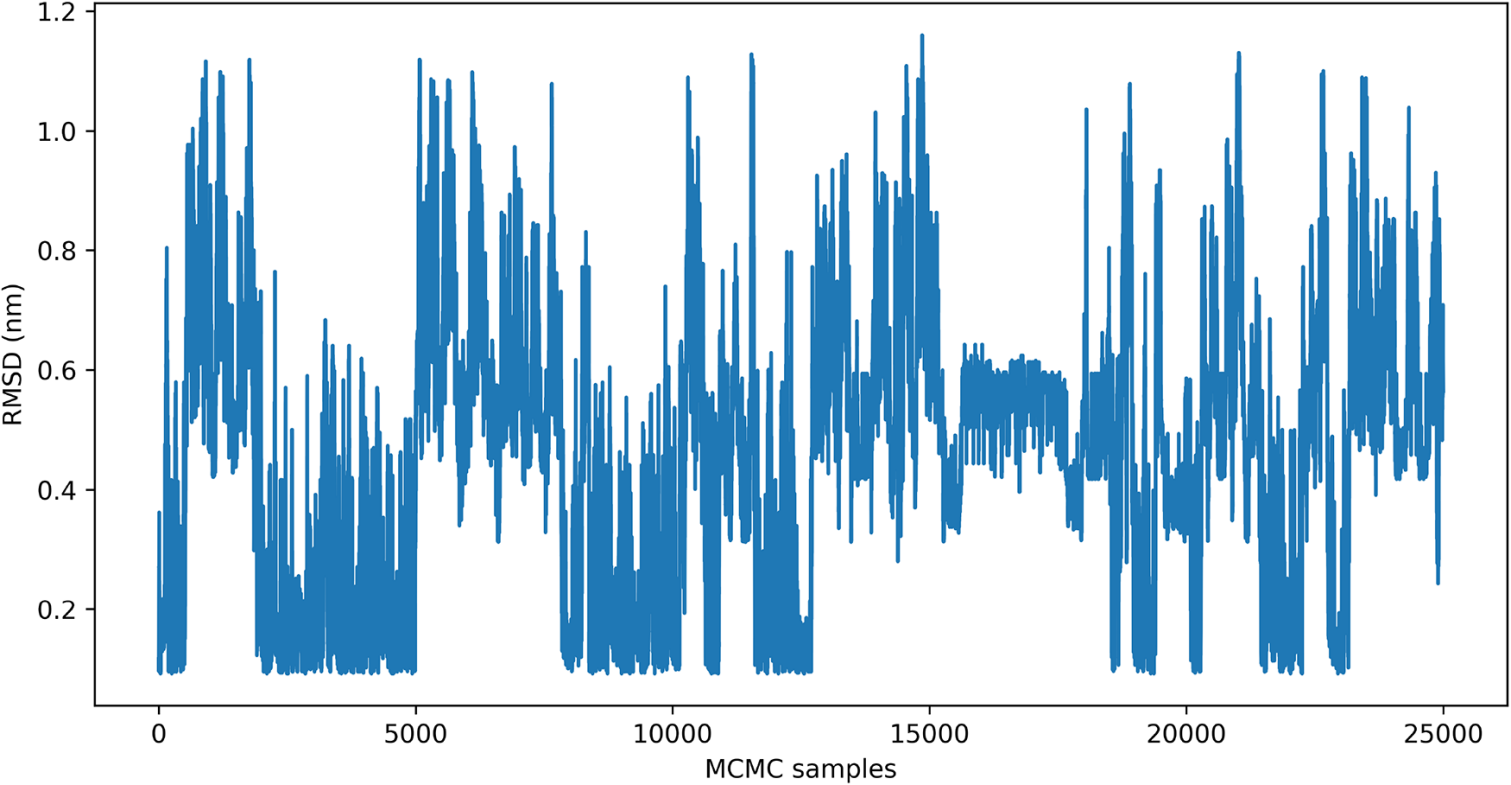
Cα-RMSD from reference structure of TrpCage structures sampled during MCMC simulation.

**Figure 8:**
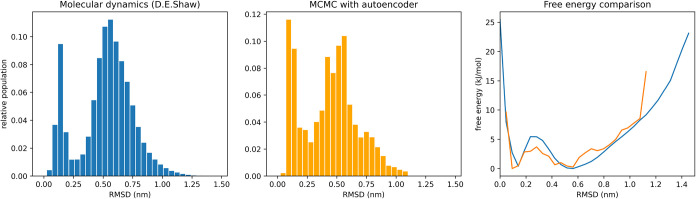
Left: Relative populations of structures with various Cα-RMSD from native structure in reference MD trajectory.^5^ Center: Relative populations of structures with various Cα-RMSD from native structure in conducted MCMC simulation. Right: Comparison of free energy surfaces calculated from the relative populations as a function of Cα-RMSD from reference PDB structure, with reference in blue and conducted MCMC in orange.

**Figure 9:**
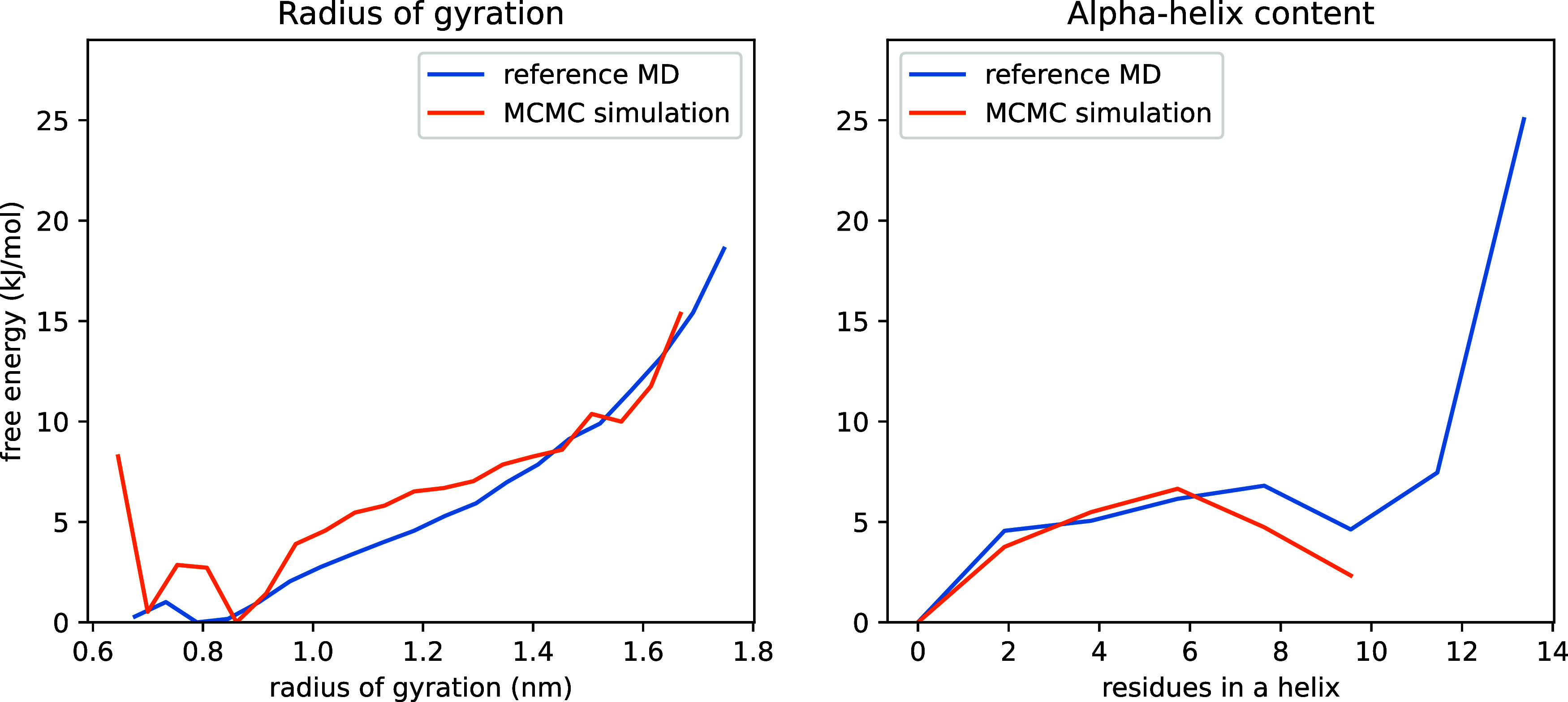
Left: Comparison of free energy surfaces as a functions of radius of gyration. Right: Comparison of free energy surfaces calculated as a functions of α helix content.

### 3.2 Nonfolding W6F-TC5b-TrpCage

The autoencoder for the W6F-TC5b-TrpCage miniprotein was trained as described in the Section 2. As for the TrpCage system, the autoencoder successfully reversibly reduces the dimensionality of the miniprotein conformations. Figure 10 depicts structures from the training data set projected into the 2D latent space by the trained encoder and selected conformations of the miniprotein decoded from hand-picked coordinates from this latent space.

**Figure 10:**
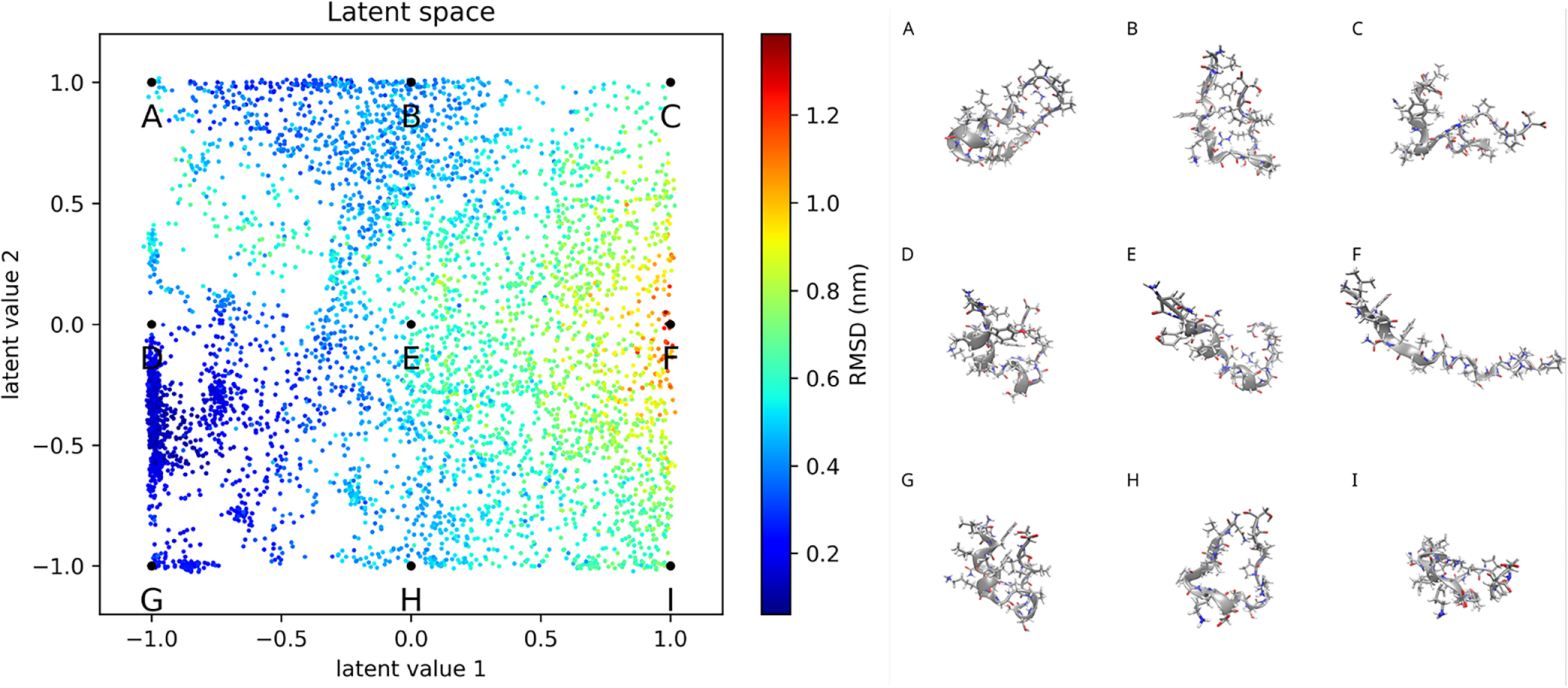
Left: Latent space of W6F-TC5b-TrpCage structures encoded by trained model. Color depicts Cα-RMSD of the encoded protein structures from the folded reference TrpCage structure. Right: W6F-TC5b-TrpCage structures decoded from hand-picked coordinates from the latent space depicted by letters A–I.

Prior to the MCMC, 4096 W6F-TC5b-TrpCage conformations were calculated by the decoder. The inputs were latent space coordinates on a 64 × 64 grid ranging from −1.25 to 1.25. Structures had their potential energy minimized in an implicit solvent as described in the Section 2. The potential energies of the prepared conformations are visualized in Figure 11. MCMC simulation was conducted as described, with parameters *f* = 400 and *d* = 0.0003, starting from the conformation homologous to folded structure of TrpCage. The temperature for MCMC was set to 290 K, the same temperature as used by the D. E. Shaw Research MD simulation of TrpCage,^5^ to allow for a comparison of the MCMC samples to the published MD trajectory and the MCMC samples of TrpCage (see above). Ten million Metropolis-Hastings sampling attempts were conducted. In total, 4,253,714 structures were accepted, so the acceptance ratio was approximately ∼42%. Since samples were collected in every *f*-th frame, this resulted in a 25,000 samples. The sampling covered all relevant areas of the latent space (see Figure 11) and the system also several times briefly visited the area of LS with structures homologous to the folded conformation of the TrpCage (see Figure 12).

**Figure 11:**
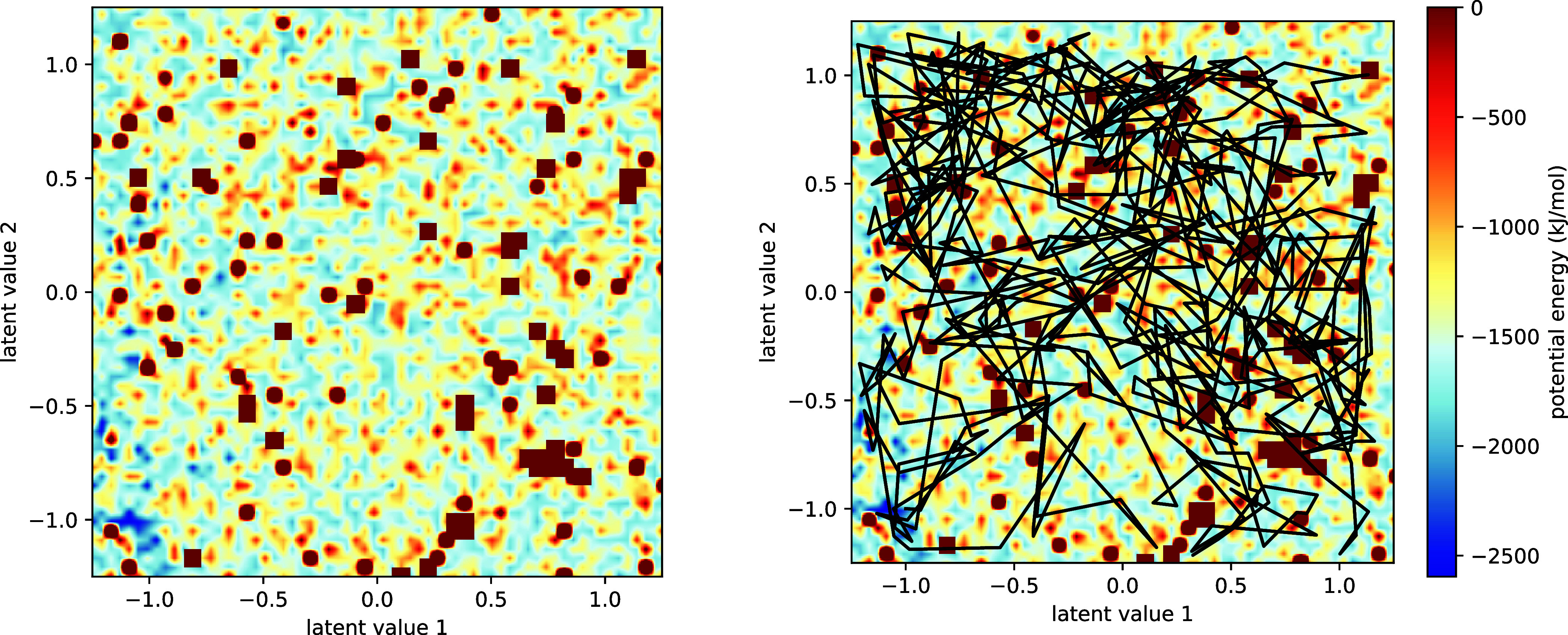
Left: Interpolated potential energy surface of W6F-TC5b-TrpCage latent space. Right: MCMC samples visualized over the latent space. Only every 50th frame is shown for better clarity.

**Figure 12:**
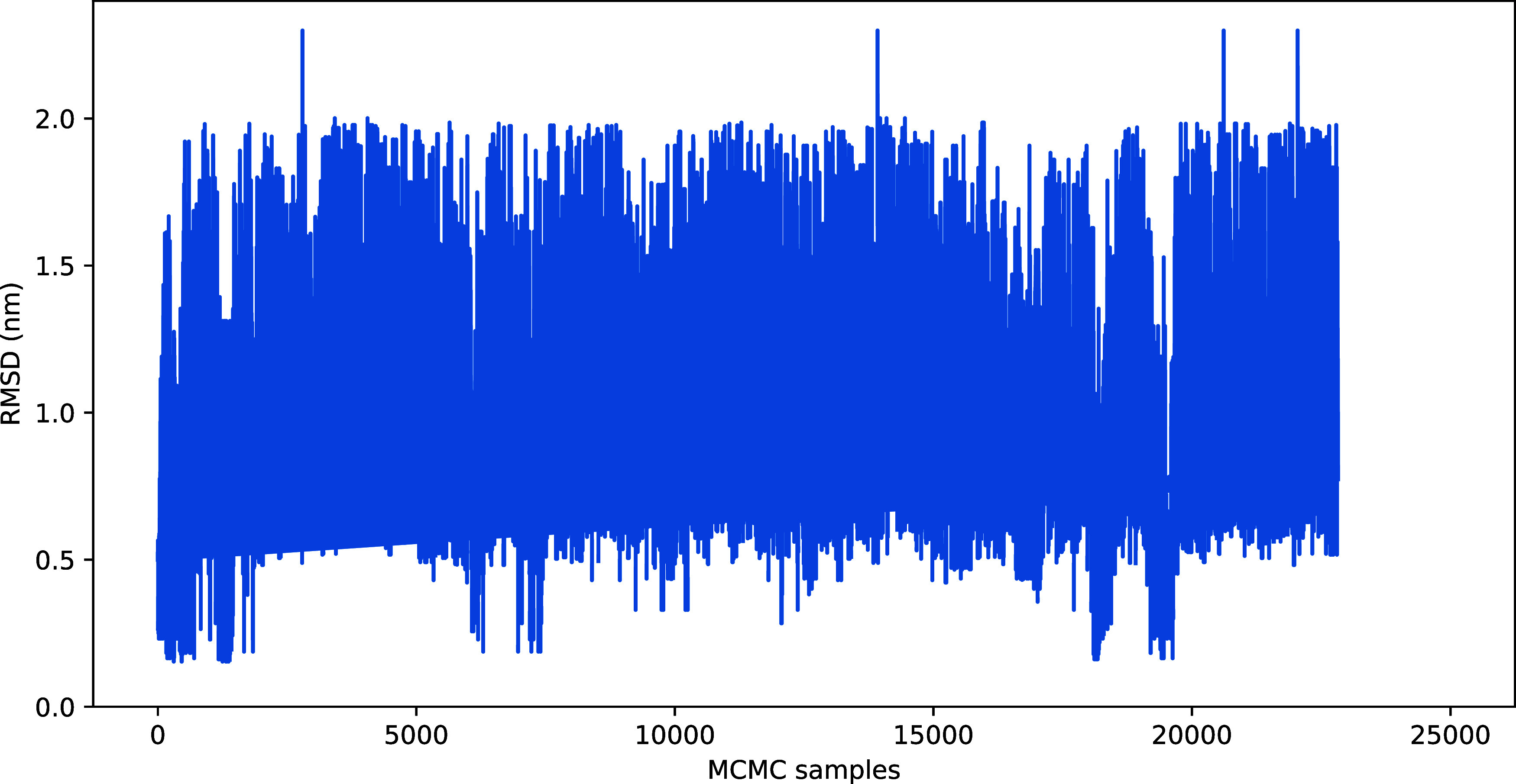
Cα-RMSD from reference structure of W6F-TC5b-TrpCage structures sampled during MCMC simulation.

We compared the relative populations of structures based on their Cα-RMSD from the reference structure in the conducted MCMC simulation with populations in the long MD simulation published by the D. E. Shaw Research.^5^ The Cα-RMSD values can be compared because the values were calculated from the same reference structure and same 20 Cα atoms of both homologous miniproteins. The comparison of the free energy surfaces is visualized in Figure 13 and it shows that the MCMC simulation workflow correctly models that the “fold” of the nonfolding W6F-TC5b-TrpCage is unstable.

**Figure 13:**
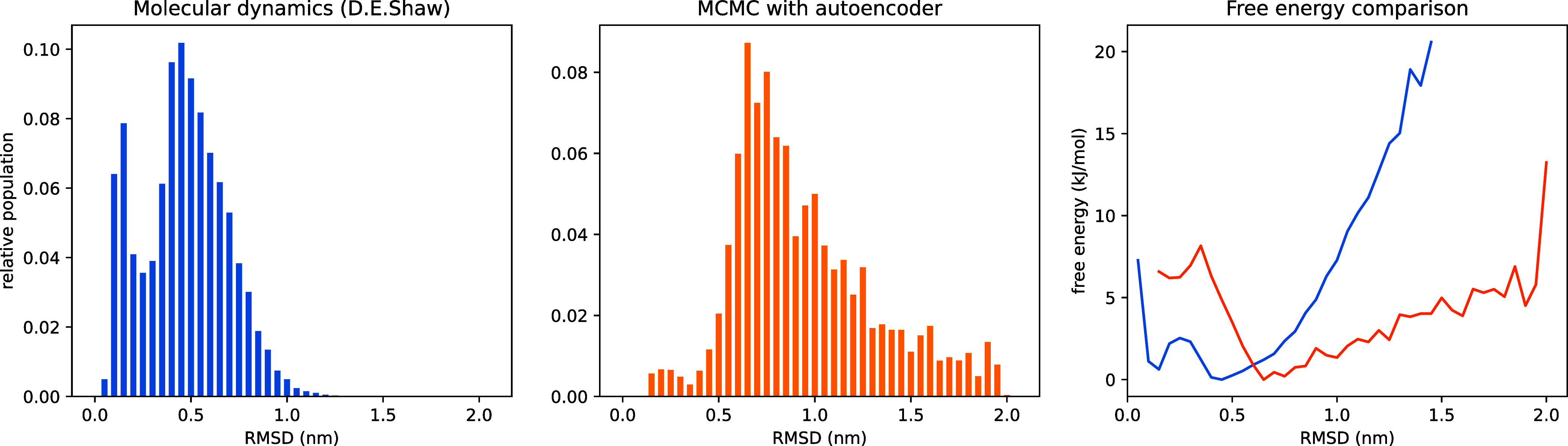
Left: Relative populations of structures with various Cα-RMSD from reference structure in reference MD trajectory of K8A-TC10b-TrpCage variant.^5^ Center: Relative populations of structures with various Cα-RMSD from the reference structure in conducted MCMC simulation of W6F-TC5b-TrpCage variant. Right: Comparison of free energy surfaces calculated from the relative populations, with reference MD of the folding K8A-TC10b-TrpCage variant in blue and conducted MCMC of the nonfolding W6F-TC5b-Trpcage variant in orange.

### 3.3 Villin Headpiece

The autoencoder for the villin headpiece miniprotein was trained as described in the Section 2. As for the TrpCage system, the autoencoder successfully reversibly reduces the dimensionality of villin conformations. Figure 14 depicts structures from the training data set projected into a 2D latent space by the trained encoder part of the model. The selected conformations of the miniprotein decoded from hand-picked coordinates from the latent space are also presented in Figure 14.

**Figure 14:**
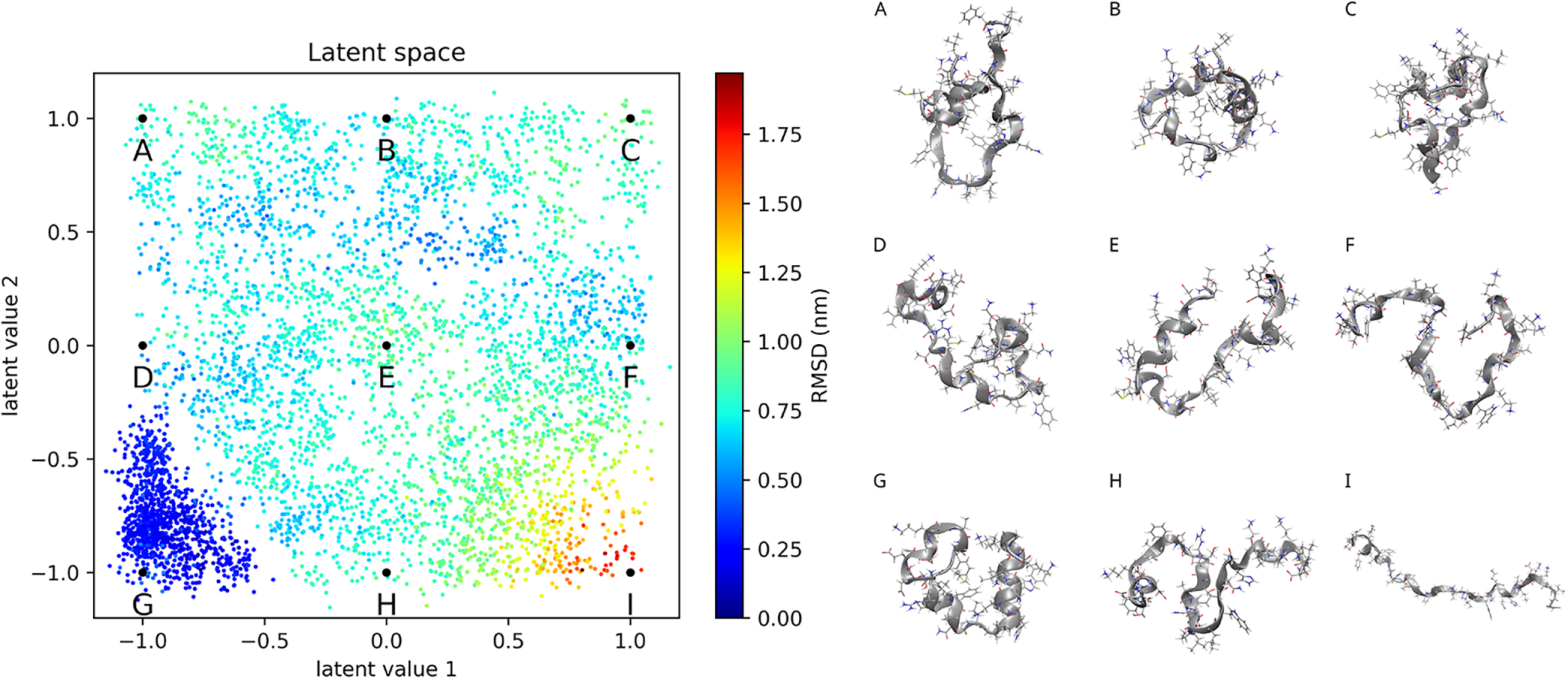
Left: Latent space of Villin structures encoded by the trained model. Color depicts Cα-RMSD of the encoded protein structures from the folded reference PDB structure. Right: Villin structures decoded from hand-picked coordinates from the latent space depicted by letters A–I.

Prior to the MCMC, 4,096 Villin headpiece configurations were calculated by the decoder. The inputs were latent space coordinates on a 64 × 64 grid ranging from −1.25 to 1.25. Structures had their potential energy minimized in an implicit solvent as described in the Section 2. The potential energies of the prepared conformations are visualized in Figure 15. MCMC simulation was conducted as described, with parameters *f* = 1000 and *d* = 0.0002, starting from the folded conformation of Villin. The temperature for MCMC was set to 360 K, the same temperature as used by the D. E. Shaw Research MD simulation of villin,^5^ to allow for a direct comparison of our samples to the published MD trajectory. Twenty million Metropolis-Hastings sampling attempts were conducted. In total, 11,229,253 structures were accepted, so the acceptance ratio was approximately ∼56%. Since samples were collected in every *f*-th frame, this resulted in a 20,000 samples containing five unfolding and five folding events (see Figure 16).

**Figure 15:**
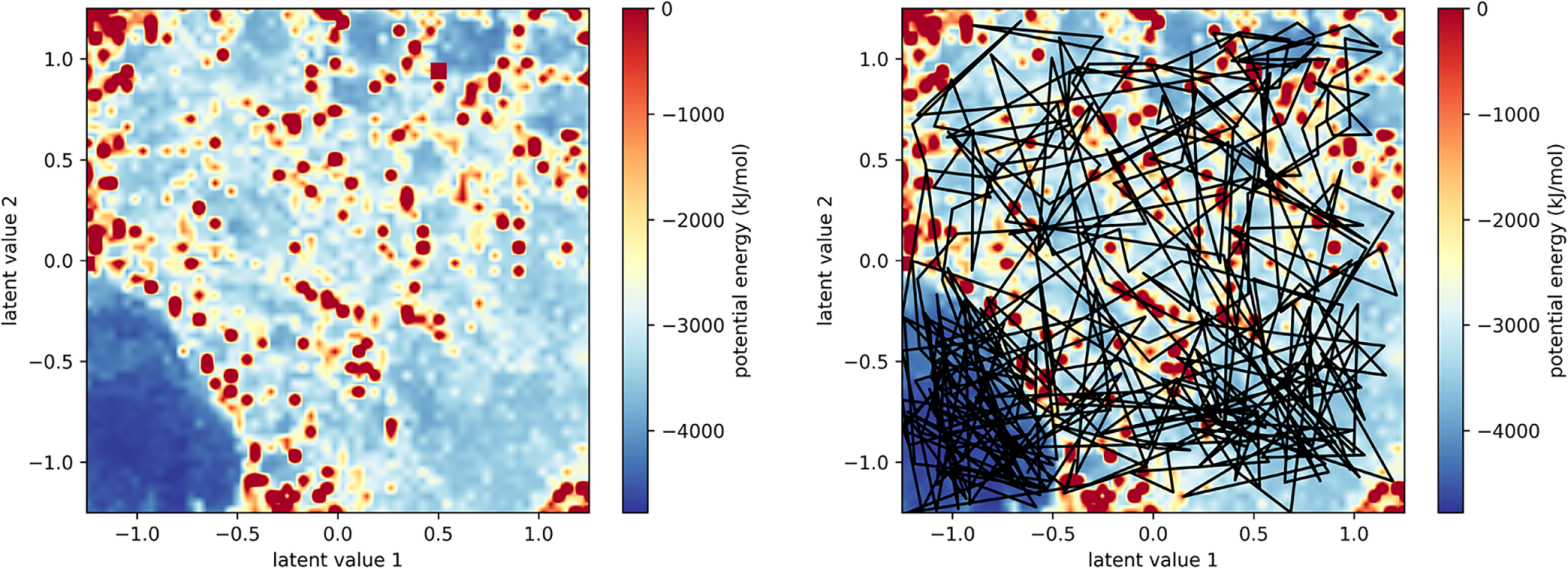
Left: Interpolated potential energy surface of Villin latent space. Right: MCMC samples visualized over the latent space. Only every 50th frame is shown for better clarity.

**Figure 16:**
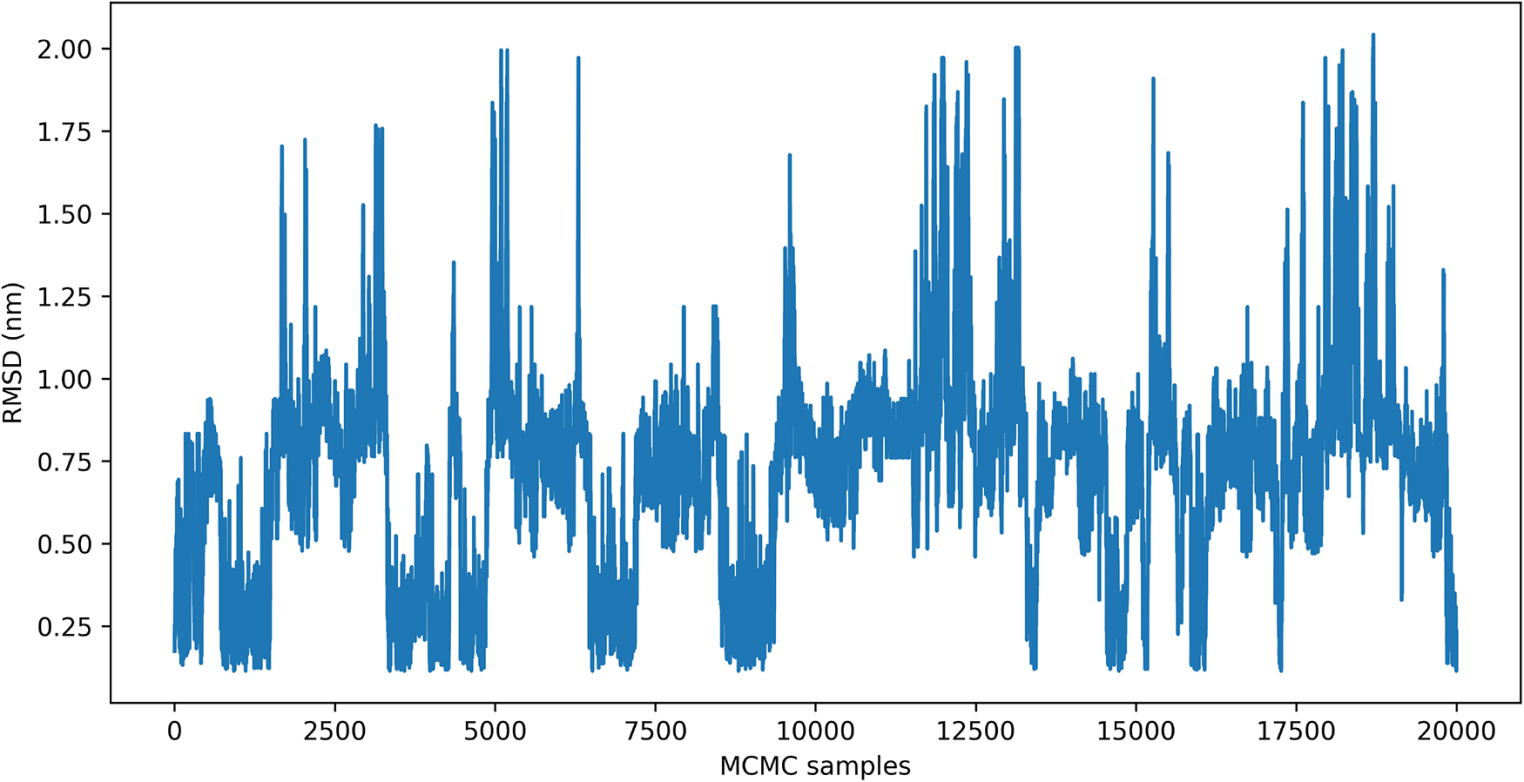
Cα-RMSD from reference PDB structure of Villin structures sampled during MCMC simulation.

We compared the relative populations of structures based on their Cα-RMSD from the reference structure in the conducted MCMC simulation with populations in the long MD simulation published by the D. E. Shaw Research.^5^ The comparison is visualized in Figure 17 and it shows that the FES from MCMC simulation is similar, but the minima and the barrier between folded and unfolded state is slightly shifted from the FES from the reference simulation. We also compared the reference and sampled populations based on their radius of gyration and α helix content (according to Dictionary of protein secondary structure (DSSP)^20^), resulting in free energy surfaces in Figure 18. We observe that both free energy surfaces from MCMC simulation take overall shape similar to the corresponding references, but differ noticeably in the areas corresponding to the completely unfolded structures, which appear to be over sampled in the MCMC. Same trend appears also in Figure 17.

**Figure 17:**
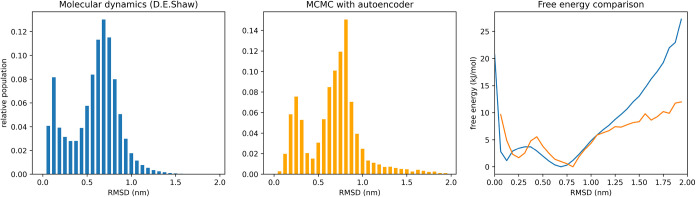
Left: Relative populations of structures with various Cα-RMSD from native structure in reference MD trajectory.^5^ Center: Relative populations of structures with various Cα-RMSD from native structure in conducted MCMC simulation. Right: Comparison of free energy surfaces calculated from the relative populations, with reference in blue and conducted MCMC in orange.

**Figure 18:**
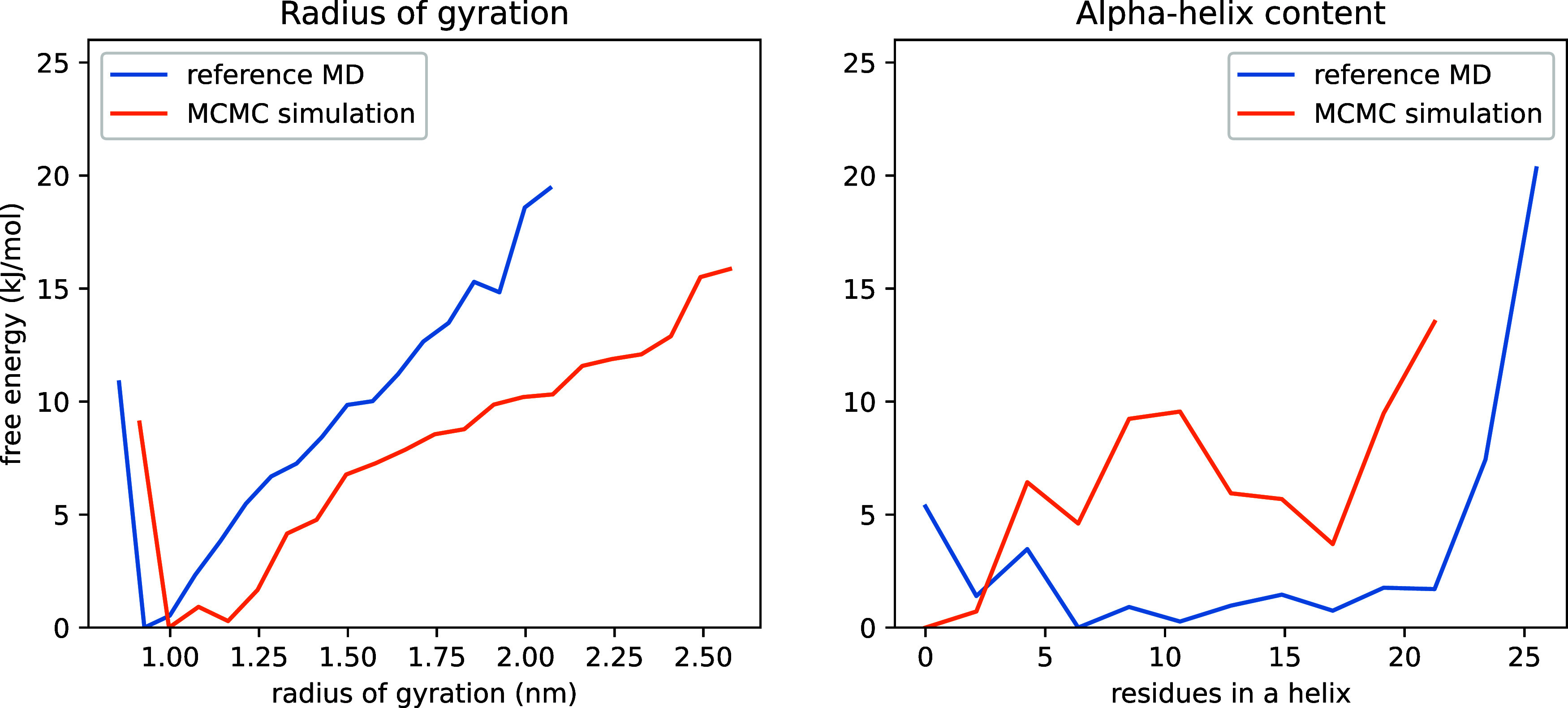
Left: Comparison of free energy surfaces calculated as a functions of radius of gyration. Right: Comparison of free energy surfaces calculated as a functions of α helix content.

### 3.4 PDZ Domain

The autoencoder for Human β-2-syntrophin PDZ domain was trained as described in the Section 2. The autoencoder successfully reversibly reduces the dimensionality of all atom protein conformations of this system. Figure 19 depicts structures from the training data set projected onto a 2D latent space by the trained encoder part of the model. The selected conformations of the miniprotein decoded from hand-picked coordinates from this latent space are also presented in Figure 19.

**Figure 19:**
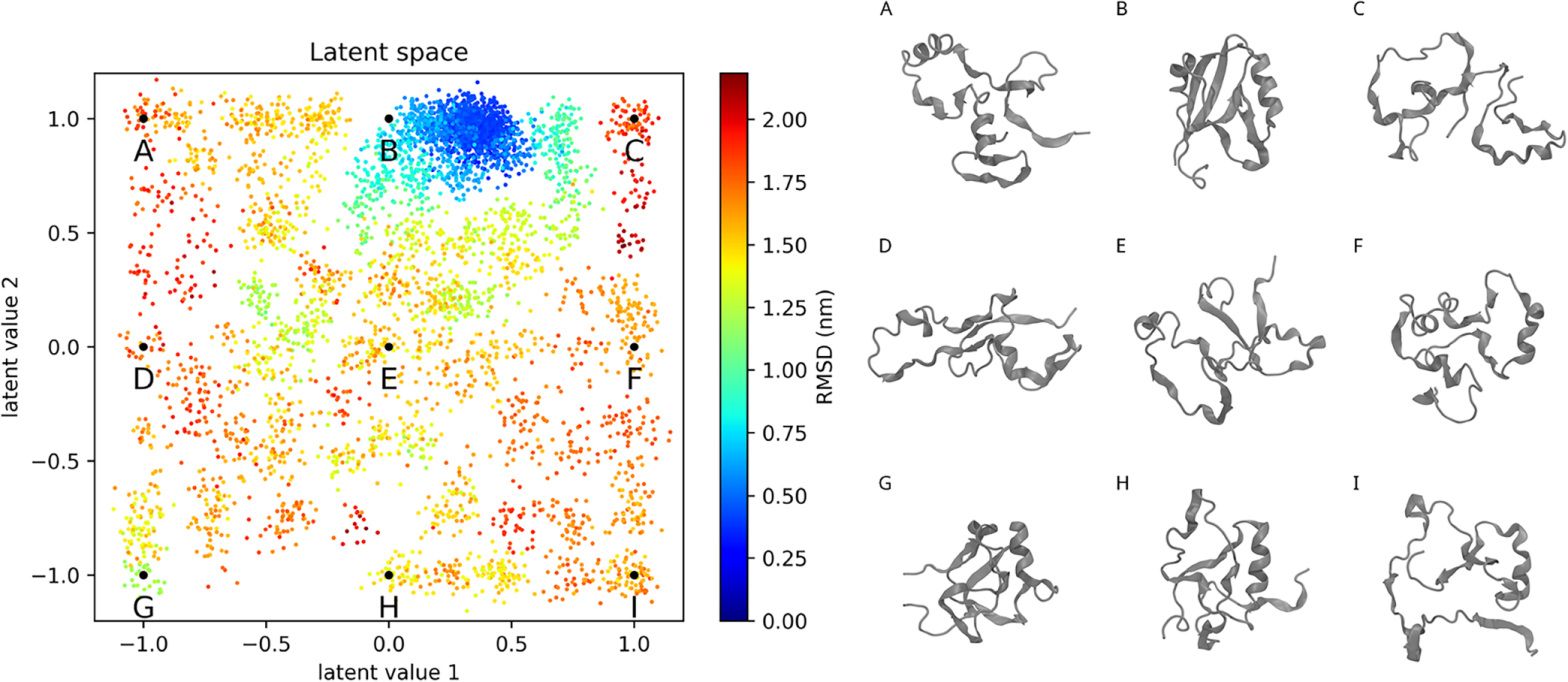
Left: Latent space of PDZ domain structures encoded by trained model. Color depicts Cα-RMSD of the encoded protein structures from the folded reference PDB structure. Right: PDZ domain structures decoded from hand-picked coordinates from the latent space depicted by letters A–I.

Prior to the MCMC, 4,096 conformations of the PDZ domain were calculated by the decoder. The inputs were latent space coordinates on a 64 × 64 grid ranging from −1.25 to 1.25. Structures had their potential energy minimized in an implicit solvent as described. The potential energies of the prepared conformations are visualized in Figure 20. MCMC simulation was conducted as described in the Section 2, with parameters *f* = 1500 and *d* = 0.00018, starting from the folded conformation. The temperature for MCMC was set to 300 K. 50 million Metropolis-Hastings sampling attempts were conducted. The MCMC sampling is visualized in Figure 20. In total, 16,598,951 structures were accepted, so the acceptance ratio was approximately ∼33%. Since samples were collected in every *f*-th frame, this resulted in 33,334 frames that contained 8 semiunfolding and 8 folding events (see Figure 21).

**Figure 20:**
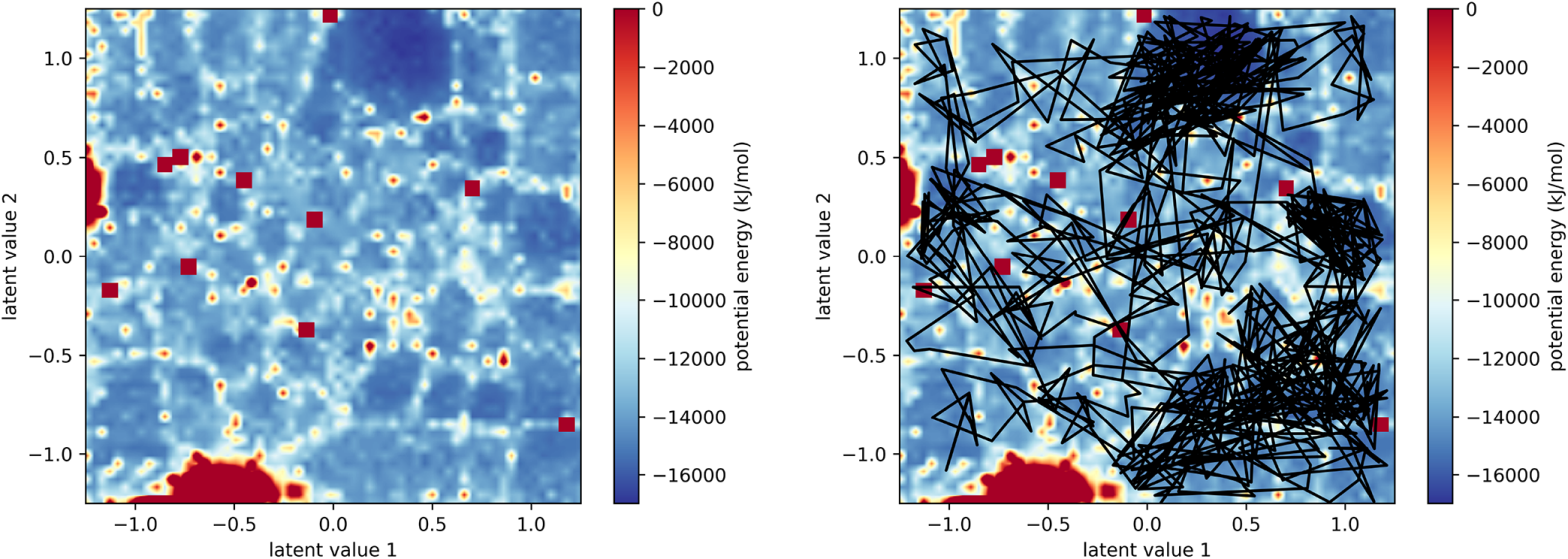
Left: Interpolated potential energy surface of PDZ domain latent space. Right: MCMC sampling visualized over the latent space. Only every 50th frame is shown for better clarity.

**Figure 21:**
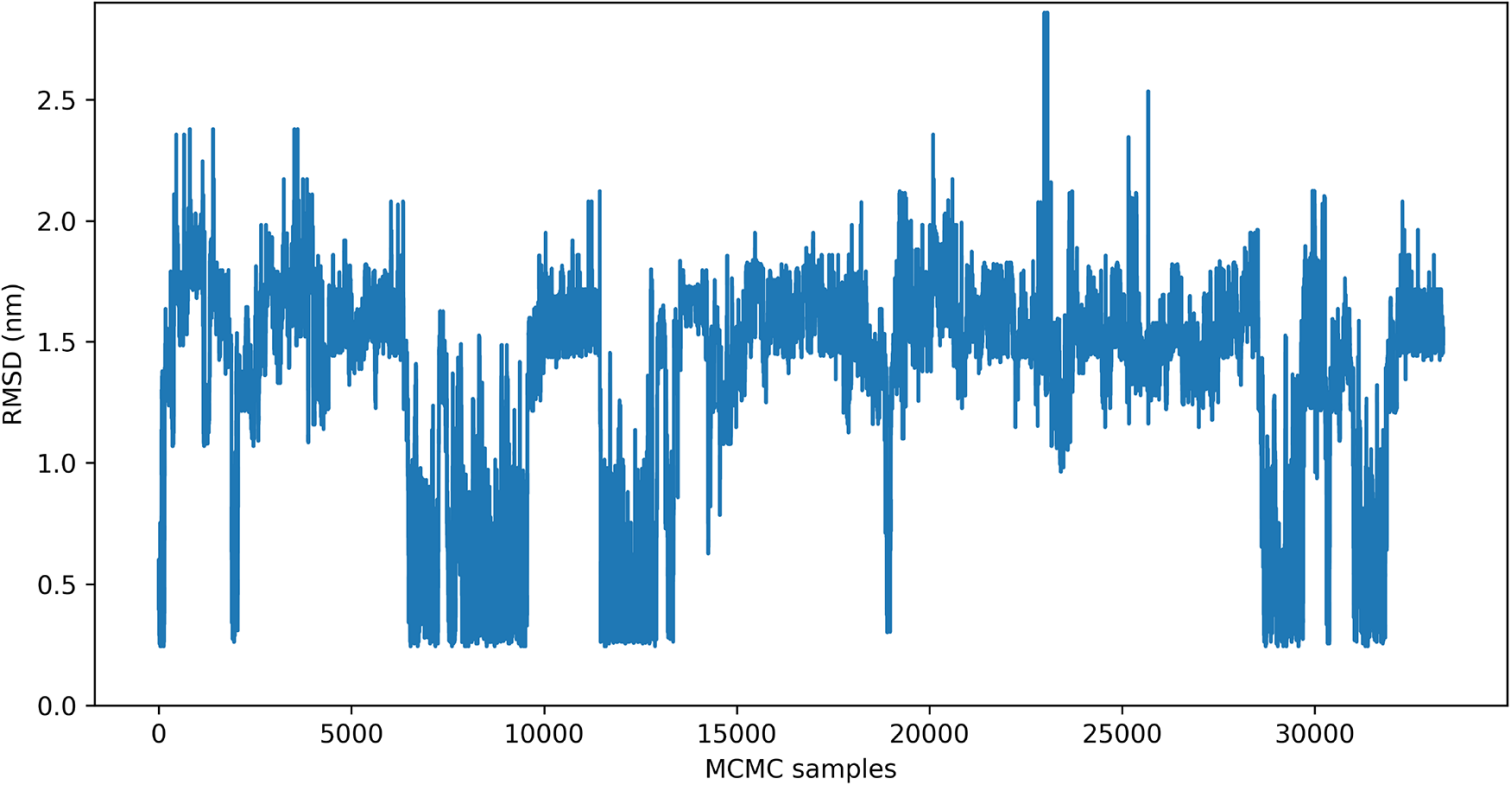
Cα-RMSD from reference PDB structure of PDZ domain structures sampled during MCMC simulation.

The free energy surface was calculated based on the Cα-RMSD of the sampled PDZ structures and is presented in Figure 22, but was not compared to a reference folding trajectory. In this case, the semiunfolded minimum was modeled as the more stable, indicating limits of the method on larger systems.

**Figure 22:**
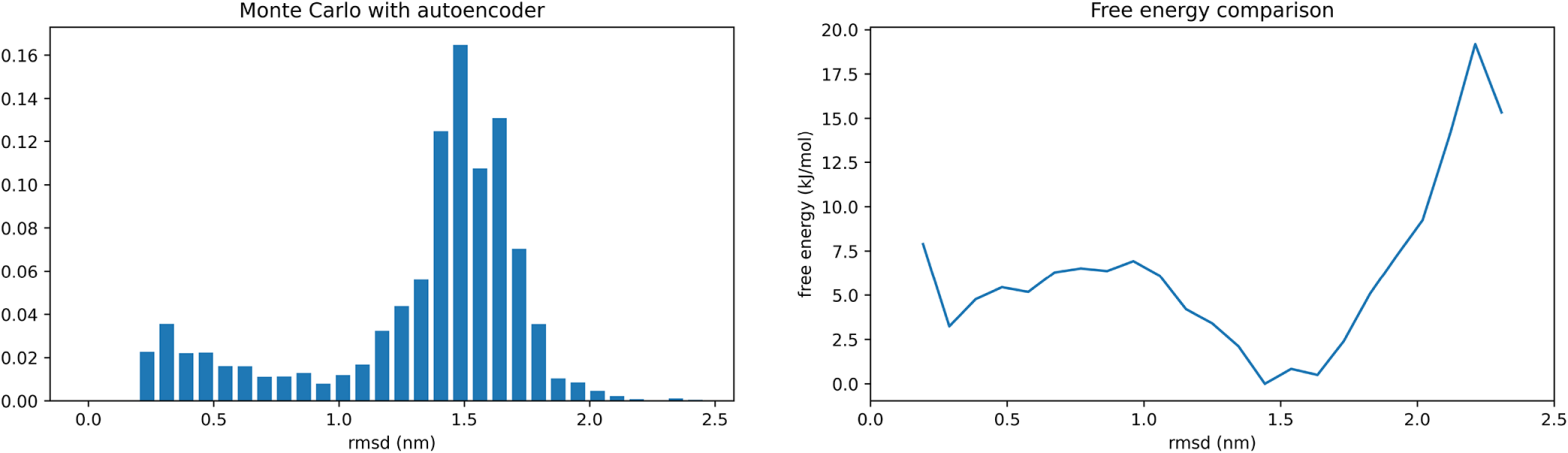
Left: Relative populations of structures with various Cα-RMSD from native structure in conducted MCMC simulation. Right: Free energy surface calculated from the relative populations of states with same Cα-RMSD from reference structure.

We tested the effect of various hyperparameters of the method to elucidate the reasons behind the observed failure. Change of the f and d parameters did not change the result significantly, besides changing the speed of its convergence or LS exploration. Also, changing the implicit solvent used in the energy calculations did not result in a significant improvement. Increasing the amount of training data also did not have a significant effect on the MCMC simulation, although AE training converged slightly faster.

We observed that the encoded structures in the LS of the PDZ domain system (Figure 19) were distributed into distinguished clusters, sometimes without transitions between neighboring clusters and with high potential energy barriers between the corresponding local minima. This was not observed in the LS of smaller systems. We believe that the high potential energy barriers caused the MCMC simulation to often get stuck in the LS, which influenced the resulting free energy surface.

This is likely caused by the autoencoder failing to efficiently encode a continuous path from the folded state to every misfolded state in the 2D latent space. We assume that a more-dimensional LS would be more successful in encoding not only the individual conformations but also a continual and realistic path between them. However, employing a more dimensional autoencoder would necessitate a significant overhaul of the method presented here, especially the amount of the protein structures decoded prior to MCMC simulation would quickly grow beyond practical with the number of LS dimensions. One option for the workflow improvement could involve precalculation of only the relevant areas of the more-than-two-dimensional LS during MCMC simulation on-the-fly. We plan to research this direction of possible improvements to the method in the near future.

In an attempt to make the LS less restricting for the AE while staying in 2D, we decreased the magnitude of the random noise added to the encoded structures during training, drawing the random numbers from a normal distribution with variance 0.02 instead of 0.05 which was used for the smaller systems. With this change, each distinct conformation effectively occupied less area of the LS, which, as we hypothesize, could make the AE more efficient at encoding such a high-dimensional system. After this change in AE training, an MCMC simulation was conducted with parameter *f* = 1500 (same as original) and *d* = 0.0001 with 50 million MCMC attempts, resulting in 33,335 samples. The acceptance ratio was 50.92%. Most of the relevant areas of the LS were visited at least once, as visualized in Figure 23. We observed a free energy surface (Figure 24) with the folded and misfolded minima with almost the same free energy value, achieving a result corresponding to the reality to a significantly higher degree than the previous MCMC run.

**Figure 23:**
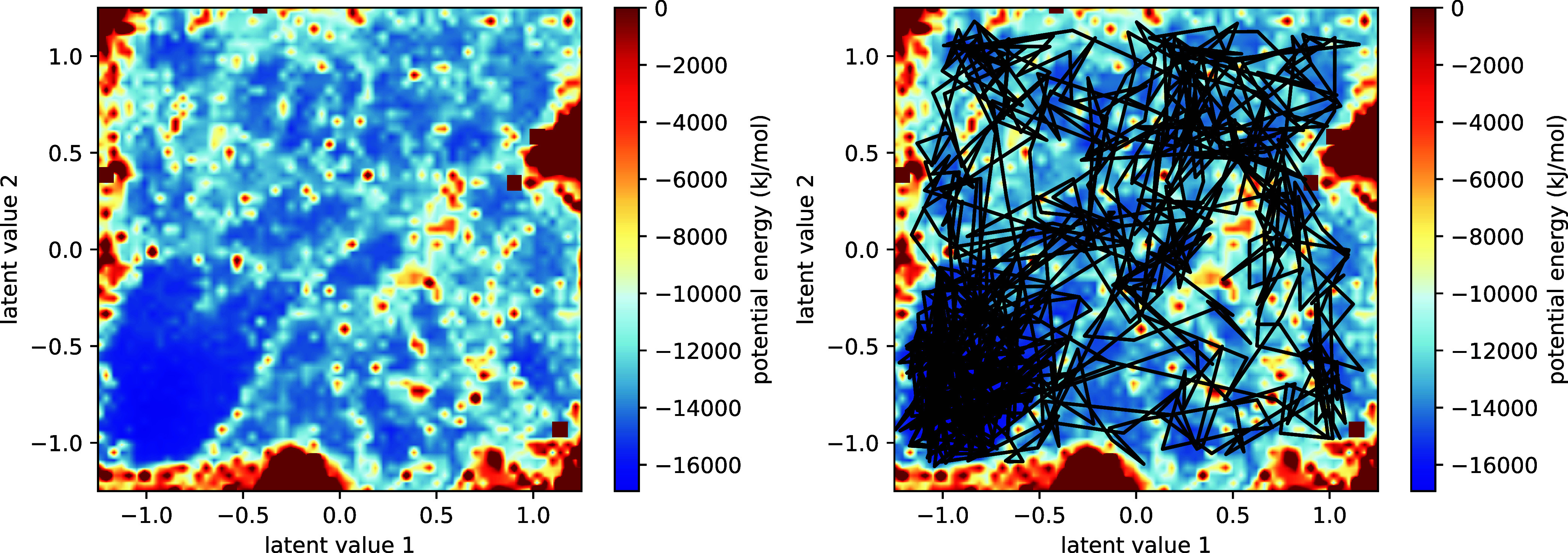
Left: Interpolated potential energy surface of PDZ domain latent space after retraining the autoencoder with noise = 0.002. Right: MCMC sampling visualized over the latent space. Only every 50th frame is shown for better clarity.

**Figure 24:**
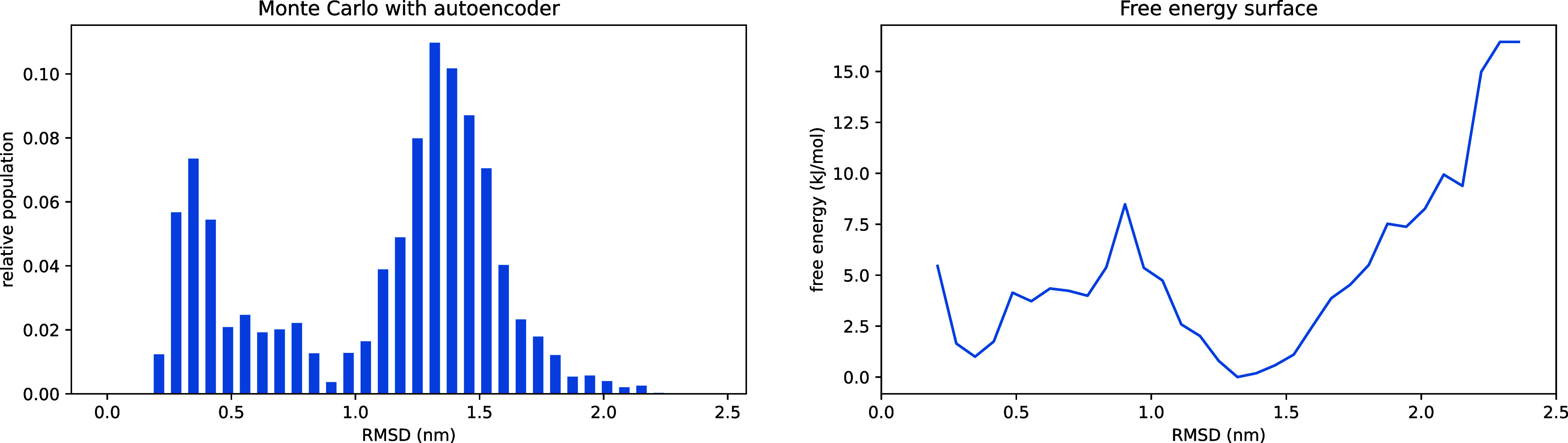
Left: Relative populations of structures with various Cα-RMSD from native structure in conducted MCMC simulation. Right: Free energy surface calculated from the relative populations of states with same Cα-RMSD from reference structure.

To summarize these findings, we admit that the 2D LS is not an optimal choice for modeling the folding and unfolding of proteins with the size similar to the 95-residue PDZ domain. Although such calculations can be performed, we recommend to approach the results with caution and validate them using experimental results or utilizing one of traditional simulation methods. We believe, however, that a 2D LS would be sufficient for modeling systems of comparable size to the PDZ domain or even larger, when applied to less challenging conformational changes than full unfolding and refolding, like opening/closing of loops or lids or quaternary structure rearrangements. The method presented here can be easily adapted to study such events by training the AE on trajectories containing appropriate data.

### 3.5 Computational Performance

We observed that MCMC simulations were very efficient in sampling diverse conformations of the studied proteins in a reasonable computational time. The MCMC simulations presented here were running at approximately 13,900, 11,100, and 9200 Metropolis-Hastings steps per second for TrpCage, Villin, and PDZ domain, respectively. Considering that protein structures were sampled every *f*th Metropolis-Hastings step, the actual performance was approximately 138, 11, and 6 samples/second for TrpCage, Villin, and PDZ domain, respectively. For these calculations, we were using a single CPU core and no GPU acceleration. The computational time was mostly spent on interpolating potential energies from precalculated values and calculating the Metropolis-Hastings acceptance probability for each MCMC step. The performance could possibly be further improved by utilizing batch MC, introduced by Klein et al.^22^

Of course, the performance analysis described above considers only the final MCMC simulation, which requires some calculations to be run in advance. The wall times required for these steps are as follows based on our observations.

The preparation of the training data set requires running MD simulations at high temperature to sample structures of the studied protein, which takes from few hours to few days of running GPU-accelerated MD on a small HPC cluster. For the TrpCage system, our wall times were 4 × 9 h (run in 4 parallel jobs) on machines using 8 CPU cores and NVIDIA A100 GPU. Processing the MD trajectory data into a data set takes a minute. Training the autoencoder can take up to a few hours when using a single GPU for acceleration. The total wall times required for training and fine-tuning were 10, 18, and 28 min for the TrpCage, Villin, and PDZ systems respectively, when using 1 CPU core and NVIDIA A40 GPU. The fine-tuning steps involve calculations of all pairwise distances between individual atoms, so the memory requirements of this step scale quadratically as the number of atoms in the system grows (*O*(*N* ^2^)). Specifically, the training process allocated 1.2, 1.6, and 4.8 GB of GPU memory for the TrpCage systems, Villin headpiece, and PDZ domain, respectively. Sampling structures from latent space by decoder only takes a minute, but the following potential energy minimization of the decoded structures can take up to a few hours using a single GPU for acceleration. In the case of TrpCage, minimizing the 4096 structures took 43 min of wall time, i.e., around 0.6 s per structure, using single CPU core and NVIDIA A40 GPU. The Villin headpiece and PDZ domain systems were minimized in 1.7 and 7.8 s per structure, respectively. Lastly, the MCMC simulation can take minutes to hours, depending on the required number of samples.

## 4 Discussion

The method presented here is part of a broader family of latent space (LS) simulation methods whose common core idea is to employ dimensionality reduction techniques to reduce the complexity and computational cost of calculating molecular simulations. These methods resemble enhanced sampling methods based on collective variable (CV), such as metadynamics,^23^ that drive the evolution of a system in a low-dimensional CV space. However, LS simulation methods, to some extent, avoid the drawbacks of general CV-based methods, for example, their dependence on prior knowledge necessary to design effective CVs. With the recent development of machine learning (ML) models, it is no surprise that they have been intensively used in this area.

One of the recently developed methods is Timewarp by Klein et al.,^22^ which, in short, uses an ML algorithm to draw samples from the Boltzmann distribution of small molecular systems. The authors reported that their ML model is transferable to molecular systems unseen during training, which is a great achievement. Our method does not exhibit this kind of transferability, mostly because of the nature of the autoencoder working with Cartesian coordinates, where each system requires a new ML model to be trained before it can be used. However, our autoencoder-based MCMC method can be applied to larger molecular systems. We show a good performance on proteins from tens of amino acid residues up to almost one hundred (the PDZ domain used in this work has 95 amino acid residues), and it seems that the method would work for even larger systems. For the method presented here, the limiting factor in terms of system size is the GPU memory required for the autoencoder training. The number of parameters of our autoencoder architecture scales roughly quadratically with the number of atoms simulated, which increases the GPU memory requirements during training. This limitation could be addressed by employing coarse-graining, allowing for modeling the C-α atoms only if there is interest in simulating much larger systems. Unlike our method, TimeWarp with an ML model that is transferable between systems was shown on systems limited to four amino acid residues only. For larger systems, our method could be used as an alternative, before more advanced transferable methods are developed, especially when, even though special training of the new ML model is required for each new system, it is also not too computationally expensive, taking several hours of wall time when using GPU acceleration.

Recently, the family of transferable generative methods was expanded by BioEMU developed by Lewis et al.^24^ BioEMU is a transferable ML model used for sampling equilibrium ensembles of monomeric proteins. The authors report that the BioEMU inference can reach thousands of structures generated per hour on a single GPU. Some of many advantages that BioEMU has compared to the method presented here are transferability, ability to model larger proteins and overall performance. Some advantages that the method presented here may have compared to BioEMU is that the production part of the workflow, i.e., MCMC sampling, does not require a GPU. That may be an advantage for users who do not have access to GPUs on a daily basis, as they can pretrain the autoencoder and minimize its generated structures first and move to a non-GPU environment to finish the workflow. Other modest advantages of our method are that the workflow is not directly dependent on the availability of homologous sequences and that the MCMC simulation may be calculated at various temperatures of interest, while BioEMU supports only 300 K at the moment. Another difference is that the method presented here generates all-atom structures of studied protein, while BioEMU generates ensembles of Cα atoms first and the side chains may be later calculated separately.

Another state-of-the-art LS simulation method was presented by Sidky et al.,^25^ who report the development of a workflow with multiple ML models for (1) encoding featurized representation of the studied system to LS coordinates, (2) propagation of the LS representation to new LS coordinates, and (3) generative ML models to get back to molecular representation. The authors show that these models are capable of producing long trajectories that can be used to predict both the thermodynamic and kinetic parameters of the systems studied at a fraction of the computational cost of traditional MD. Sidky et al. reported that their method is capable of extrapolating to new transition paths but remains largely interpolative regarding the generation of structures unseen in training data. Both of these statements are true also for the method presented in this work as it is also data-driven.

One of the differences between the method presented here and the method developed by Sidky et al. is the architecture of the ML models used. In the method presented here, the generation of protein structures is done by a simple dense neural network transforming LS coordinates to the protein conformation space. Sidky et al. use more sophisticated generative adversarial networks, which have advantages such as the ability to generate more diverse structures from a set of input LS coordinates. A disadvantage of the more sophisticated network may be, to an extent depending on the protein being modeled, the requirement for larger amounts of samples used as training data. Another disadvantage may be potential instability of the training of the adversarial networks, which need to be trained in a carefully controlled way so that the performance of one would not exceed the other too much. On the other hand, we note that the training of the autoencoder presented here was very stable and predictable for every system size we worked with. The encoder part of the architecture developed by Sidky et al. is also designed to learn kinetic models from the training data.

On the other hand, this constrains to an extent the possible ways the users of the method can use to generate the training data, meanwhile, the users of our method may generate the training samples from a different ensemble than the production one. The respective disadvantage of the method presented here is that it learns to encode each structure independently, which may limit how efficiently the transitions between metastable states are modeled. This may also explain the results observed in PDZ domain free energy surfaces (Figures 22 and 24).

We note that Sidky et al. used training data containing the whole 208 μs long MD trajectory from the D. E. Shaw Research, while we used a smaller data set which is much easier to obtain. Our approach using thermal unfolding to sample diverse conformations may be suboptimal with respect to the ergodicity of the training data set, but for application of the method to real-world systems, it is more computationally affordable. The difference in training data can have an influence on the quality of the dimensionality reduction learned by the AE and therefore on the MCMC simulation results, for example, in Figure 17 it can be seen that the FES from our MCMC simulation follows the reference FES closely, but not perfectly. We argue that the approach shown in this work is much more computationally affordable considering the combined time needed to both generate the training data set and run the MCMC simulation itself, while providing results that reasonably correspond to the reference MD simulation data.

To quantify how efficient is the method presented here at generating novel miniprotein conformations not present in the training data, we calculated pairwise Cα-RMSD between each structure from the training data set of TrpCage and the 4096 structures generated on the grid by the decoder. For each structure in the training data set, we calculated which structure on the LS grid is the most similar and noted the Cα-RMSD value for the pair. We found that the median Cα-RMSD between a structure from the training data set and the one generated by the model is ≈0.33 nm and the maximum such value was ≈0.56 nm. We note that these values are slightly larger than corresponding values for the comparison of reference MD trajectory and the training data set, suggesting that the autoencoder does generate slightly more diverse structures than it was trained on.

One of the most well-known methods related to LS simulations are Boltzmann Generators (BG) developed by Noé et al.^26^ The method presented here is, in principle, quite similar to BG in usage, as both methods allow for generating 3D conformations of proteins based on random numbers drawn from a prior distribution. Both methods use ML models trained to generate samples of a specific system and have limited transferability to other systems. Our approach is different in the fact that our autoencoder is used primarily to reduce the dimensionality of the conformational space from 3*N* (where *N* is the number of atoms) to 2, while BG samples the normal distribution per each degree of freedom independently. BGs also employ more advanced featurization of the studied system, while our model treats all atoms equally.

Noé et al. report that when training their model, one of the most computationally expensive steps was calculating the potential energies of the generated structures during the “training by energy” mode, after which the model was generating high-probability low-energy structures. We have avoided this type of calculations in our training protocol by fine-tuning the decoder parameters to minimize atom–atom distance errors and the reciprocal distance errors. Although these calculations are computationally more expensive than training to minimize mean square error of plain atom positions, they are not as expensive as calculating potential energies (at least in our implementation). These calculations, on the other hand, require a lot of memory and limit the scalability of all-atom LS simulations to some extent. We observed that the fine-tuning described in our training protocol improves the potential energies of generated structures by 10–12 orders of magnitude compared to structures before fine-tuning (see Figure 5). The final structures are not perfect, short potential energy minimization is still necessary, but overall the process is very efficient.

In the workflow presented here, the training data set is obtained by thermal unfolding of the studied protein. Thermal unfolding samples most of the relevant low-temperature states of the folding-unfolding pathway.^27^ We are not concerned by the fact that it was not sampled from the Boltzmann distribution, as the potential influence of this is addressed by using the Metropolis-Hastings MCMC sampling. The comparison of the training data set sizes shown in Figure 4 suggests that the data set size of 50,000 samples seems to be a well-working compromise between performance and the computational cost of the training, at least for systems of comparable size. Models trained on the data set of 50,000 structures offer almost the same performance as models trained on the larger data set of 210,000 structures, while models trained on the smaller 10,000 data set suffer significant performance loss. The same conclusions are reached when comparing the quality of the structures reconstructed by the decoder, as shown in Figure 5.

We are aware that directional sampling presented here can influence the detailed balance of the sampling algorithm, therefore we analyzed the balance of the sampling algorithm empirically in the following way: we divided the latent space to discrete bins (different from the grid used for generating structures and calculating the energies) and counted transitions of the system between the bins during MCMC simulation. Flux matrix was calculated from the transition data. If the detailed balance holds (the null hypothesis), the flux matrix is symmetrical; we tested this hypothesis statistically using the χ^2^ test. The rejection/nonrejection of the null hypotheses on the significance level of 5% depends on the coarseness of the grid and the resulting flux matrix: for grids with resolution 16 × 16 or finer, the null hypotheses are rejected, for grids of 15 × 15 bins or coarser, the null hypotheses are not rejected. As the amount of data grows—when analyzing more MCMC samples—the *p*-values of the tests goes up, making it unclear if the detailed balance for finer grids is broken because of insufficient sampling or potential bias in the sampling algorithm. To interpret these findings, we conclude that the detailed balance of the sampling algorithm is not guaranteed; however, on the global scale of the latent space, the sampling behaves as balanced. We admit that in an ideal case, the workflow should not need the directional sampling scheme, but in our experience, standard MCMC sampling leads to acceptance ratios so small that the sampling is too inefficient to be practically useful.

To demonstrate the difference in sampling using the classical MCMC and the directional sampling introduced here, we tested two methods: one using a random size of steps made in the LS and one with a fixed size of steps. The first one is equivalent to the classical Metropolis MCMC sampling, with the only change being that instead of changing randomly positions of particles, the LS coordinates were changed during the generation of new attempts. The second approach using a fixed-size step is equivalent to our directional scheme with parameter *f* = 1. Since the direction of each step is independent of the direction in the last step, the sampling is balanced because for any forward move, a reverse attempt is equally likely. We ran both simulations for a total of 10,000,000 attempts. The acceptance ratios were 30 and 29%, respectively. The resulting MCMC samples for both schemes and comparison of resulting free energy surfaces are presented in Figure 25. Both methods produced nearly the same result, unfortunately, none of the known-to-be balanced sampling methods could sample the LS efficiently. Using a sampling method that is known to be balanced is, of course, preferable, and we believe that with a sufficiently well-trained AE model and well-structured LS, it could be more efficient to use. We advise users of this method to try running the method presented here first with *f* = 1. If such sampling is not efficient enough and the user is willing to use a method that may not satisfy detailed balance on fine-grained scale, the *f* parameter can be set to higher values to enhance the sampling.

**Figure 25:**
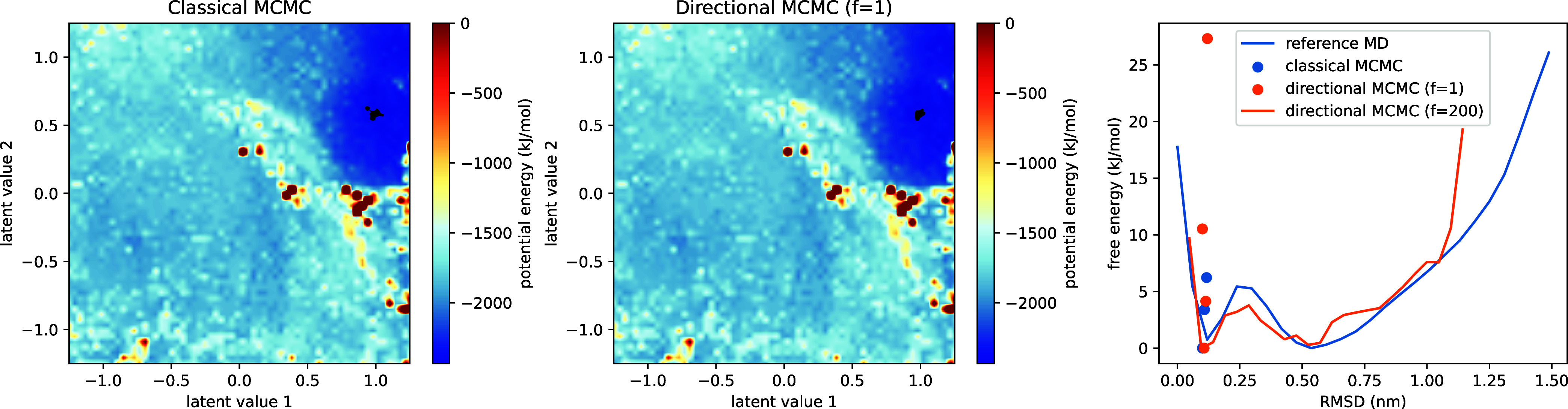
Comparison of balanced sampling methods applied on TrpCage LS. Left: Classical Metropolis MCMC samples visualized over the potential energy surface of the LS. Center: Samples from the directional sampling scheme presented here, with parameter *f* = 1, visualized over the potential energy surface of the LS. Right: Comparison of free energy surfaces calculated from the reference MD trajectory (blue line), by Metropolis MCMC sampling (blue dots), by the directional sampling with *f* = 1 (orange dots) and by directional sampling with *f* = 200 as presented here in the Results (orange).

Another potential weak point of the method presented here could be copying the distribution of structures found in the training data set, if the MCMC sampling would have copied the distribution of structures found in the training data set, without providing any other benefit than sampling randomly from this memorized distribution that was created at much higher temperature than what the MCMC simulation runs at. To show that this is not the case in our method, we present two free energy surfaces in Figure 26, one prepared by encoding all training data set structures, counting them into 2D histogram over the LS and calculating a free energy from the relative populations in the bins, and a second free energy surface calculated analogously but from the actual samples from the MCMC simulation of the TrpCage system. The differences in the two graphs show that the method presented here does not just copy the populations of samples found in the training data but provides new information based on the data it was trained on. If the two figures were identical or very similar, it would instead indicate that the MCMC simulation only reproduces the training data, which were sampled at different conditions.

**Figure 26:**
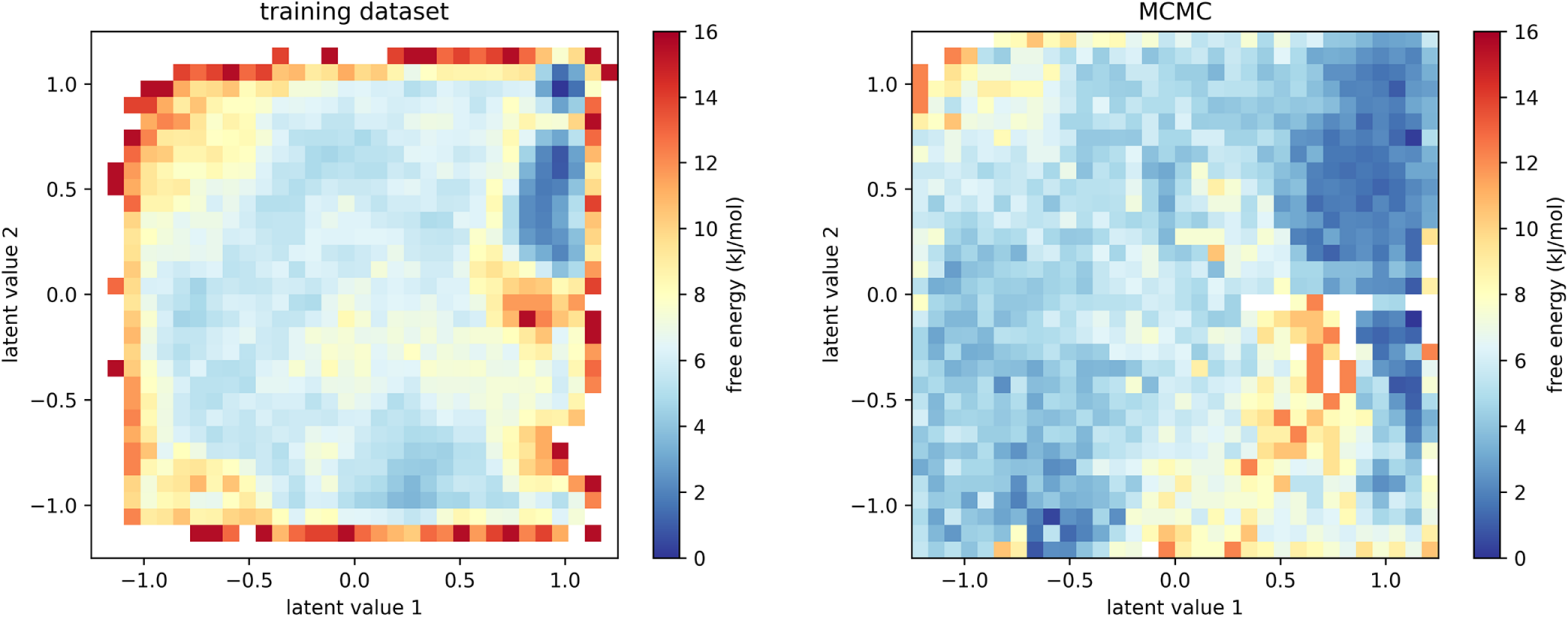
Left: Fictitious free energy surface on latent space that would have emerged, if the MCMC sampling worked poorly and only followed population of samples from training data. Right: Actual free energy surface calculated from the MCMC samples. Both pictures are related to the TrpCage MCMC simulation presented in this work.

The dimensionality of the latent space was set to 2 for two reasons: second for easy visualization purposes, but more importantly, the ergodicity of the MC simulation requires that the MC samples the same state repeatedly and aperiodically, i.e., after a nonfixed and finite number of steps. It is known that in unlimited 2D space, a random walker is expected to visit the initial state after a finite number of steps, but for dimensionality >2, this is not guaranteed. While this does not directly apply in our case, as the latent space is not unlimited, but it is confined to interval (−1.25, 1.25)^2^, we still recommend limiting the dimensionality of the latent space to 2–3. Furthermore, even though the LS is numerically limited, the number of different encoded structures is in principle infinite. The more conformations are encoded in the LS, the smaller individual steps would be used for the random walk, and the more the simulation would be similar to the case of unlimited 2D plane. It is also known that metadynamics, which also acts in a low-dimensional CV embedding of a studied system, works best in 2D, less well in 3D, and usually works poorly in more dimensions without special treatment, e.g., in bias exchange metadynamics or related methods. Lastly, using 2 LS dimensions allows us to decode all of the structures in the LS in advance of the MCMC simulation, allowing the computational speedup presented here.

A unique sampling technique employed in the method presented here is MCMC sampling of the potential energy surface without explicitly calculating the corresponding structures. Before MCMC, protein conformations and their energies in the implicit solvent were precalculated in discrete bins over the AE latent space. However, the difference in potential energy of two similar structures can be high, making the sampling of the high-energy structures computationally expensive (i.e., requiring many MCMC attempts). Thus, we ran MCMC on a continuous potential energy surface interpolated from the discrete energies calculated in the implicit solvent. On this continuous potential energy surface, the sampling efficiency can be adjusted using the parameters *f* and *d*, as described in the Section 2. We believe that this sampling technique does not lead to an alteration of the resulting free energy surface calculated from the MCMC samples but only enhances the computational efficiency of the method. To support this claim, we tested three different grid resolutions and compared the results in the SI. It can be argued that the linear interpolation used for the continuous potential energy surface does not have physical meaning on its own; however, the latent space itself is already a result of nonlinear interpolation of the training data, which the autoencoder learns without supervision. Our method can be seen as coarse-graining on the energy level. An analogy of this approach can be seen in how protein conformational dynamics is modeled using normal-mode analysis by Bahar et al.,^28^ since normal-mode analysis is another example of potential energy surface coarse-graining. Arguably, as an alternative or in combination with this interpolated energy sampling, batch MC could be used, as described by Klein et al.^22^

We assume that the obstacle of finding high potential energy differences for very similar protein conformations arises mostly from the implicit solvent potential energy calculation, which does not involve hydrogen bonding between protein and individual water molecules. This can disadvantage the potential energy of unfolded protein structures to some extent, as they contain less protein–protein hydrogen bonds, which are not replaced directly by interactions with explicit solvent. We work on addressing this issue by including an explicit solvent in the structures for MCMC sampling, but so far this is a work in progress. We acknowledge Noé et al.^26^ for inspiring our research in this direction.

To compare the computational efficiency of the method presented here to traditional MD, we can count the number of folding and unfolding events observed in the MCMC samples presented in results and in corresponding MD trajectories from literature, as the MCMC samples do not have a time scale in the traditional sense. We observe that the TrpCage MCMC simulation sampled 10 and 9 unfolding/folding events, respectively, which is comparable to the 12 and 12 unfolding/folding events in D.E.Shaw’s 208 μs MD simulation. The Villin headpiece MCMC simulation sampled 5 unfolding and 5 folding events, which is somewhat less than in the reference MD simulation (34 and 34 of both folding and unfolding events). From these comparisons, we can conclude that MCMC sampling can be very efficient at sampling the folding and unfolding events of the studied miniproteins, and that the results of the MCMC simulations presented here are comparable to the respective reference MD.

Presented comparisons of free energy surfaces from this method and from reference MD simulations show that the method models free energy surfaces correctly within the chemical accuracy of 4.18 kJ/mol; however, the errors seem to slowly increase with system size when applied to modeling miniprotein folding. Traditional simulation methods like MD can reach higher precision of predictions than the method presented here; however, delivery of such results requires orders of magnitude more computational time. Similar applies to modern state-of-the-art enhanced sampling methods involving machine learned collective variables, like the ptl-tSNE by Hradiská et al.^29^ or, with various computational requirements, to adaptive sampling approaches, like the RAVE by Ribeiro et al.^30^

The kinetic parameters, like height of the free energy barrier between folded/unfolded local minima, are predicted within chemical accuracy of 4.18 kJ/mol when compared to known ground-truth values for TrpCage and Villin headpiece systems. For TrpCage, it is slightly underestimated while for Villin headpiece system it is at slightly higher Cα-RMSD value. The errors probably arise from multiple sources: first, the potential energies calculated for generated structures are calculated in implicit solvent GBn2, which does not involve interactions with explicit water molecules that might be important for modeling the miniprotein folding processes. Another source of errors may be the dimensionality reduction, which is learned by the autoencoder without supervision and may, in principle, under-perform in important areas of the latent space where transitions occur. The dimensionality reduction also in principle limits the diversity of distinct structures a deterministic decoder can model, but on the other hand, it allows for higher computational performance and interpretability of the latent space.

A small part of the discrepancy between the reference and our free energy surfaces can be explained by force fields used, as we have used the Amber14 ^16^ force field to model protein atoms, while D.E.Shaw used the CHARMM22* force field in their work.^5^ We believe that the different force field can have a small influence on the results presented here; however, other factors like the dimensionality reduction or the implicit solvent do play a bigger role. To quantify the effects, we constructed the potential energy surface of the TrpCage system LS using the CHARMM22*^31^ force field used by D. E. Shaw and we compared it to the one calculated with Amber14. The comparison in Figure 27 shows the CHARMM22* potential energy surface subtracted from the Amber14 potential energy surface. The comparison shows that some individual structures, especially on the potential energy barriers, tend to be minimized differently using the two force fields discussed here, but the overall potential energy surface is very similar.

**Figure 27:**
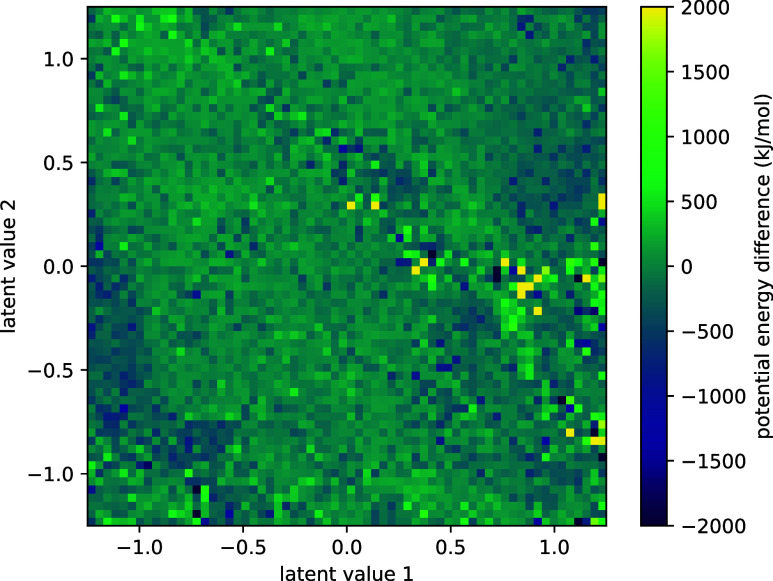
Visualization of the potential energy difference between structures predicted by autoencoder and minimized using force fields CHARMM22* and Amber14.

These findings confirm that the method presented here generates predictions quickly, but researchers requiring more accurate predictions of the results may want to support these results by other methods focused on kinetic predictions by design, for example, VAMPnets^32^ or time-lagged autoencoders.^33^ Other useful alternative methods include machine-learning-based methods focused on CV design for enhanced sampling, for example, parametric time-lagged tSNE,^29^ RAVE,^30^ REAP,^34^ or DeepDriveMD.^35^

To assess the accuracy of thermodynamic predictions, we note that the method correctly predicts the nonfolding W6F-TC5b-TrpCage variant to not be stable in the structure homologous to the folded TrpCage structure; however, for the PDZ domain, it predicts the nonfolded minimum to be slightly more stable. Therefore, for systems of comparable size to the 95-residue PDZ domain, we suggest the method presented here to be used for less demanding tasks, like modeling allosteric behavior, sampling diverse structures close to native structure for follow-up studies, for example, for small-molecule docking. Other notable alternative uses of the method can be the design of efficient collective variables for molecular processes of interest, where researchers can use the insight gained from the dimensionality reduction or MCMC trajectory, or any other use cases preferring computational performance and interpretability.

Lastly, our method is focused on training the AE and its subsequent use for MCMC sampling. A common drawback of BG, our method and other data- driven methods is that the ergodicity of the resulting trajectories depends a lot on the quality of the training data, as discussed above. The limitations of our method were in part already described and include limited ability to extrapolate far beyond training data, limited transferability and scalability to very large systems. Most of these drawbacks are shared with some other methods from the LS simulation family. In future work, our method could be extended to include explicit solvent after implementing the permutational invariance of solvent molecules as explained by Noé et al.^26^ Another topic for potential future research is extension of the method presented here to simulations of protein complexes (similarly to the method presented by Jones et al.^36^) or protein–ligand interactions, combining the MCMC sampling with other established enhanced sampling method, or involving adaptive sampling approaches.

In conclusion, we have developed a new LS simulation method based on a simple AE followed by MCMC sampling. The machine-learning model we use has a very simple architecture and training protocol compared to many other recently published methods from this family, promising its robust applicability to diverse protein systems. Special attention was paid to ensure that the model training, MCMC simulation, and other related calculations are computationally affordable in real-world use. We demonstrate a computationally efficient way to generate a training data set for our model and an efficient MCMC sampling technique capable of modeling protein conformational behavior in much shorter computational time than traditional simulation methods, while providing results of comparable accuracy.

## Supplementary Material



## Notes

Thermal unfolding protocol, potential energy minimization protocol, comparison of grid resolutions for MCMC simulations; comparison of MCMC simulations with various *f* and *d* parameters and comparison of learning curves for different data set sizes for the PDZ domain system (PDF)
