# Supplementary material for: Generative Autoencoders Coupled to Monte Carlo Simulation Allow Efficient Protein Conformation Sampling
Source: J Chem Theory Comput. 2026 May 25;22(11):5807–26. doi: 10.1021/acs.jctc.5c01813 (PMC13255177; doi:10.1021/acs.jctc.5c01813)
Supplement: Supplementary file 1 [file ct5c01813_si_001.pdf]

Supporting Information: Generative autoencoders coupled to Monte Carlo simulation allow efficient protein conformation sampling

Jan Beránek, Guglielmo Tedeschi, and Vojtěch Spiwok*

Department of Biochemistry nad Microbiology, University of Chemistry and Technology, Prague, 166 28, Czech Republic

E-mail: spiwokv@vscht.cz

1 Thermal unfolding protocol

First, the system with protein solvated in explicit water was equilibrated at a temperature of 300 K. Then, 10 ns of MD simulation were conducted at 400 K. From this simulation, four equally spaced frames were sampled and they all served as starting frames for four subsequent 50 ns long MD simulations conducted at 500 K. Before each MD simulation, the system had potential energy minimized. The temperature was kept at selected temperature using Parrinello-Bussi thermostat ([1]) and pressure coupling was conducted with Parrinello-Rahman barostat ([2]), with pressure set at 1 bar.

Then, sample frames were collected for the training dataset: the MD simulation run at 400 K was sampled at each 1 ps and the MD simulations run at 500 K were each sampled at each 5 ps. These frames were concatenated and saved for later use. 50,000 frames in total

were used as the training dataset. The miniprotein structures were superimposed on the reference structure by minimizing the Cα-RMSD.

Cartesian coordinates of all protein atoms were parsed by the mdtraj[3] package and stored as a NumPy array. The coordinates of the atoms were then scaled, so their values were between 0.0 and 1.0. They were shuffled and split, so that 80 % of them were used for training and the remaining 20 % were used as a test dataset to assess whether the system was overfitting.

We analysed whether the thermal unfolding dataset misses significant amount of conformations sampled in low-temperature reference MD by calculating pairwise Cα-RMSD between reference MD simulation of TrpCage by D. E. Shaw (sampled every 20 ns = 10,440 frames) and the 50,000 frames in the thermal unfolding dataset, resulting in the following pairwise Cα-RMSD matrix visualized in Figure S1

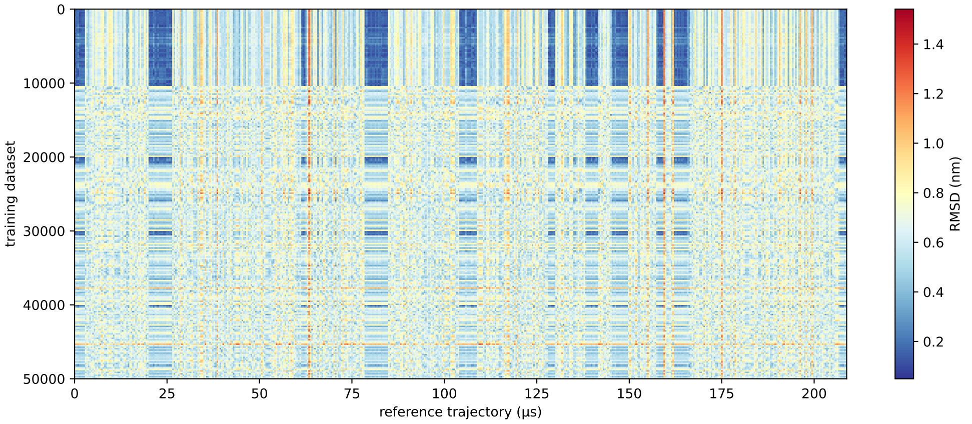

Figure S1: Pairwise Cα-RMSD between the thermal unfolding dataset and reference MD trajectory.

For each frame in the reference MD, we calculated, how distant from it is the most similar frame from the thermal unfolding dataset, by calculating the minimum Cα-RMSD value along the x axis of the matrix in the Figure S1. The maximum such value was only ≈0.38 nm and the median for the 10,440 reference frames was ≈0.26 nm. Based on these metrics, we consider the thermal unfolding dataset satisfactory and representative for the

purpose of machine learning. To put these numbers into perspective, we visualized the structure from the reference MD which is most dissimilar to its most similar sample from the thermal unfolding, where the Cα-RMSD between the structures was ≈0.38 nm. The visualization is in the Figure S2.

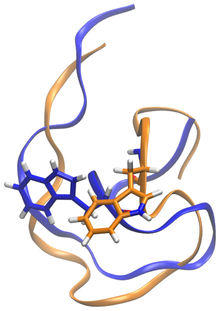

Figure S2: Comparison of the structure of TprCage from the reference MD trajectory (in orange) with the most similar structure from thermal unfolding dataset (in blue). The Cα-RMSD of the pair is 0.38 nm. For all other studied structures from the reference trajectory, there was a structure in the thermal unfolding dataset with a smaller Cα-RMSD than for the pair in the picture.

2 Potential energy minimization

Potential energy minimization was applied using two algorithms, Steepest descent with some custom changes and L-BFGS.[4,5] First, the system was minimized by the Steepest Gradient Descent (SD) algorithm implemented by us for use with OpenMM,[5] inspired by the SD implementation in OpenMMTools with the following changes: In the classical SD algorithm in GROMACS,[6] the particles of a system are moved in the direction of force acting on them by an amount set by the `step size` parameter divided by the sum of the magnitude of all forces. Then, if the potential energy of the system decreases, the new structure is accepted and `step size` increases by a certain factor (1.2 in GROMACS). If the potential energy is higher, the new structure is not accepted, and the `step size` parameter is multiplied by another factor (0.2 in GROMACS) to get smaller. These steps then continue until maximum

number of steps is reached or until the largest force acting on any particle is smaller than predefined value.

In our case, we found that for some structures generated by decoder, this approach does not converge or produces unstable results, never finding local energy minimum at all. This does not happen frequently, only in less than 1 % of cases, but when generating thousands of structures, it is nevertheless undesirable, so we made the following changes to the algorithm in order to make it more reliable: First, between each comparison of the old and new potential energy, several steps are performed with the `step size` parameter slowly increasing. This allows the system to go across some potential energy barriers on a way to a stable minimum when starting from extremely unstable configurations. Furthermore, the parameters for increasing or decreasing the `step size` parameter were set to 1.2 and 0.5.

In addition, if a new structure was not accepted after several steps, the structure was changed by artificially pushing the particles against the potential energy gradient by a very small amount.

This SD algorithm was successfully used for most of the generated structures. However, if it did not find a minimum after a certain number of steps, the initial structure was submitted to the L-BFGS minimization algorithm implemented in OpenMM,[5] which was used for very short minimization until the largest force acting on any particle in the system was lower than 10^4 kJ mol^{-1} nm^{-1}, and then its resulting structure was returned to our SD algorithm to finish the descent to the local energy minimum. We used our SD algorithm to fine-tune the minimization, because it is a deterministic algorithm, which is not true for L-BFGS due to its inherent stochastic nature. On the other hand, L-BFGS was sometimes necessary for the initial minimization of the most challenging decoder output, so our workflow included both in an attempt to "get the best of both worlds".

3 Comparison of grid resolutions for MCMC simulations

We have run three MCMC simulations of theTrpCage system using different grid resolutions to investigate the influence the grid resolution has on the simulation results. We tested grid resolutions of 256×256, 64×64 and 16×16. The other parameters used were the same for all simulations: $f = 200$ and $d = 0.007$. Each simulation consisted of 5,000,000 MCMC attempts. Resulting free energy surfaces for Cα-RMSD from TrpCage reference structure together with the reference free energy surface calculated from the D. E. Shaw long MD simulation are presented in Figure S3. The median absolute errors of the free energies predicted from MCMC simulations compared to the reference was ≈ 1.0 kJ/mol, ≈ 1.1 kJ/mol and ≈ 1.1 kJ/mol for the resolutions 256, 64 and 16, respectively. The free energy surfaces from the 256 grid and 64 grid are overall more similar to the reference surface, are both less noisy and model the two-well surface better compared to the free energy surface using the resolution 16.

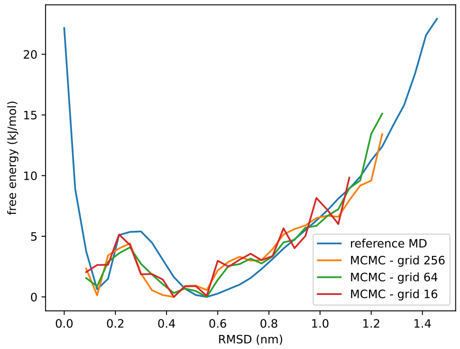

Figure S3: Comparison of the reference free energy surface of TrpCage (in blue) and free energy surfaces calculated from samples from MCMC simulations using grid resolutions of 256×256 (in orange), 64×64 (in green) and 16×16 (in red).

4 Comparison of MCMC simulations with various f and d parameters

To demonstrate the influence the values of parameters f and d have on the MCMC sampling, we ran 9 MCMC simulations of TrpCage system. The f parameters were 50, 200 or 800 and the d parameters were 0.0001, 0.001 or 0.01. Other settings were kept the same: 5,000,000 MCMC attempts and temperature 290 K. Acceptance ratios and numbers of observed folding/unfolding events for each simulation are presented in Table S1. The results are visualized as samples over the latent space in Figure S4 and as free energy surfaces based on Cα-RMSD from the reference PDB structure in Figure S5. Quick analysis of these Figures shows that the simulations containing several folding and unfolding events and with average acceptance ratio around 30-60 % also predict the best free energy surfaces from the selection.

Table S1: Acceptance ratios (AR) and number of observed folding (N_f) and unfolding (N_u) events for sample populations generated by MCMC with various f and d parameters

	$d = 0.0001$	$d = 0.001$	$d = 0.01$
$f = 50$	AR = 98 %<br>$N_f = 0$<br>$N_u = 1$	AR = 83 %<br>$N_f = 0$<br>$N_u = 0$	AR = 7 %<br>$N_f = 0$<br>$N_u = 0$
$f = 200$	AR = 97 %<br>$N_f = 0$<br>$N_u = 1$	AR = 57 %<br>$N_f = 13$<br>$N_u = 13$	AR = 5 %<br>$N_f = 0$<br>$N_u = 0$
$f = 800$	AR = 94 %<br>$N_f = 1$<br>$N_u = 2$	AR = 43 %<br>$N_f = 28$<br>$N_u = 29$	AR = 4 %<br>$N_f = 0$<br>$N_u = 0$

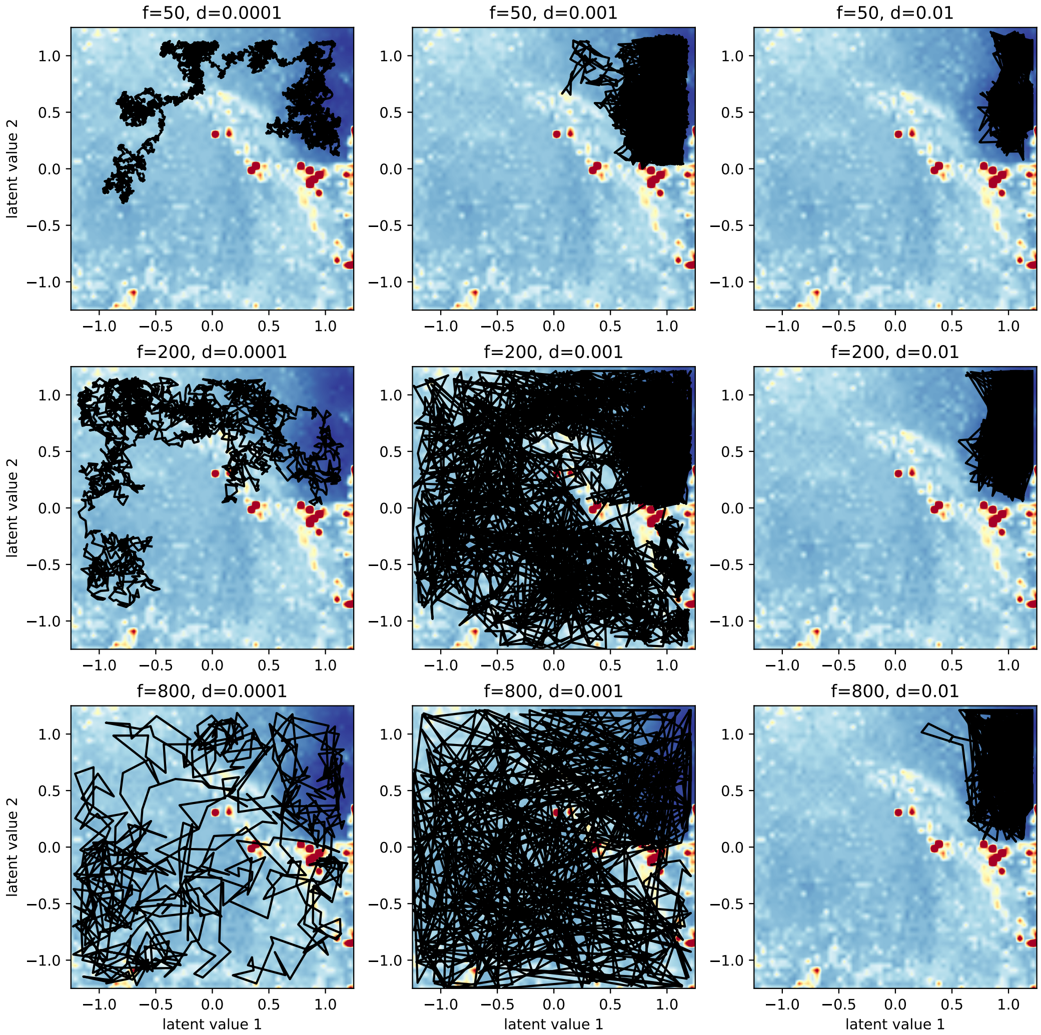

Figure S4: MCMC samples visualized on the latent space for various f and d parameters. Only every 10th sample is shown for clarity.

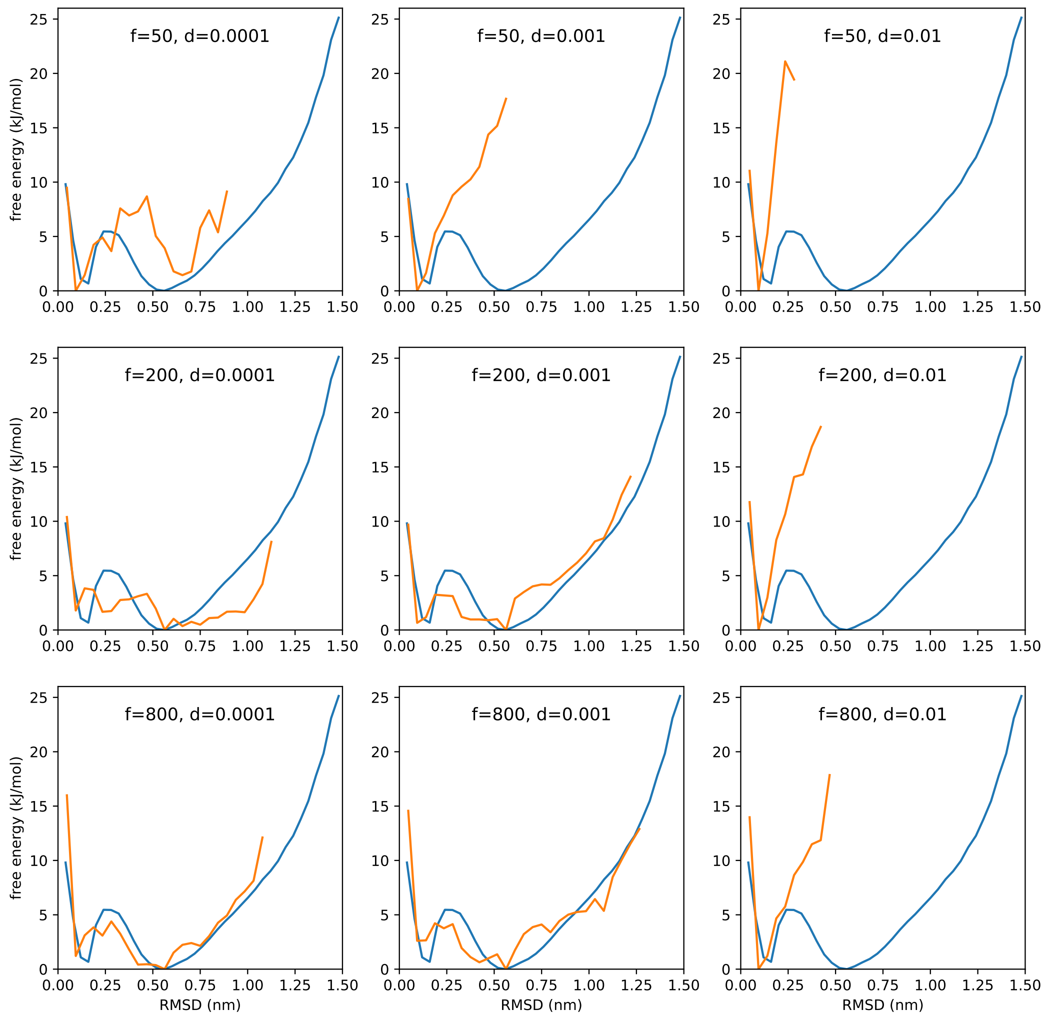

Figure S5: Comparison of free energies as functions of Cα-RMSD from the reference structure for reference simulation (in blue) and populations sampled by MCMC (in orange) for various combinations of f and d parameters.

5 Comparison of learning curves for different dataset sizes for the human β-2-syntrophin PDZ domain system.

We have tested the effect of different dataset sizes on the model training also on the PDZ domain system to see if the learning behaves differently when applied on bigger systems. The comparison was done the same way as described for the TrpCage system already described in Results: Datasets compiled from the structures sampled from the thermal unfolding trajectories of PDZ domain had sizes of 210,000, 50,000 and 10,000 structures respectively. All datasets were split into a training set (80 %) and a validation set (20 %). Learning curves for the three dataset sizes are shown in Figure S6. Quality of the structures generated by the decoder were assessed by calculating potential energies of 500 randomly selected structures from the dataset, which were encoded and reconstructed by models after each part of the training/fine-tuning process for each dataset size. The comparison of the distributions in the energies of the reconstructed structures is in Figure S7.

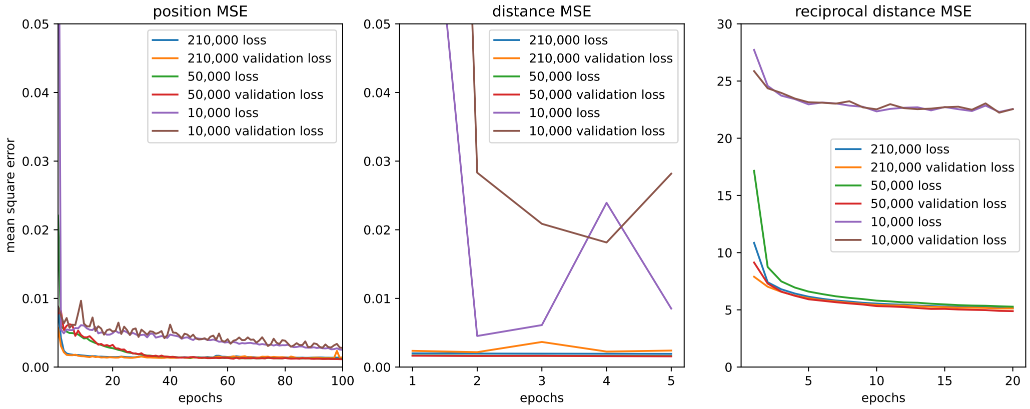

Figure S6: Comparison of learning curves for three different dataset sizes on the PDZ domain system.

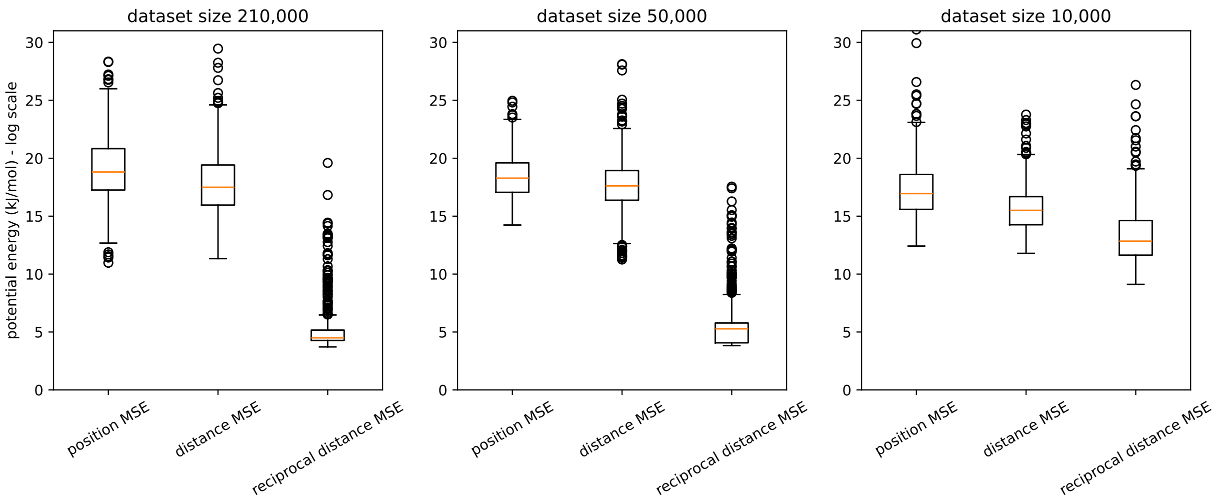

Figure S7: Distributions of potential energies of PDZ domain structures reconstructed by models trained on different dataset sizes and after different training/fine-tuning treatments.

References

(1) Bussi, G.; Donadio, D.; Parrinello, M. Canonical sampling through velocity rescaling. *J. Chem. Phys.* **2007**, *126*, 014101.

(2) Parrinello, M.; Rahman, A. Polymorphic transitions in single crystals: A new molecular dynamics method. *J. Appl. Phys.* **1981**, *52*, 7182–7190.

(3) McGibbon, R. T.; Beauchamp, K. A.; Harrigan, M. P.; Klein, C.; Swails, J. M.; Hernández, C. X.; Schwantes, C. R.; Wang, L.-P.; Lane, T. J.; Pande, V. S. MDTraj: A Modern Open Library for the Analysis of Molecular Dynamics Trajectories. *Biophysical Journal* **2015**, *109*, 1528 – 1532.

(4) Liu, D. C.; Nocedal, J. On the limited memory BFGS method for large scale optimization. *Math. Program.* **1989**, *45*, 503–528.

(5) Eastman, P.; Swails, J.; Chodera, J. D.; McGibbon, R. T.; Zhao, Y.; Beauchamp, K. A.; Wang, L.-P.; Simmonett, A. C.; Harrigan, M. P.; Stern, C. D.; Wiewiora, R. P.; Brooks, B. R.; Pande, V. S. OpenMM 7: Rapid development of high performance algorithms for molecular dynamics. *PLOS Computational Biology* **2017**, *13*, e1005659.

(6) Pronk, S.; Páll, S.; Schulz, R.; Larsson, P.; Bjelkmar, P.; Apostolov, R.; Shirts, M. R.; Smith, J. C.; Kasson, P. M.; van der Spoel, D.; Hess, B.; Lindahl, E. GROMACS 4.5: a high-throughput and highly parallel open source molecular simulation toolkit. *Bioinformatics* **2013**, *29*, 845–854.
